# Computational quantification and characterization of independently evolving cellular subpopulations within tumors is critical to inhibit anti-cancer therapy resistance

**DOI:** 10.1186/s13073-022-01121-y

**Published:** 2022-10-20

**Authors:** Heba Alkhatib, Ariel M. Rubinstein, Swetha Vasudevan, Efrat Flashner-Abramson, Shira Stefansky, Sangita Roy Chowdhury, Solomon Oguche, Tamar Peretz-Yablonsky, Avital Granit, Zvi Granot, Ittai Ben-Porath, Kim Sheva, Jon Feldman, Noa E. Cohen, Amichay Meirovitz, Nataly Kravchenko-Balasha

**Affiliations:** 1 The institute of Biomedical and Oral Research, The Hebrew University of Jerusalem, 9103401 Jerusalem, Israel; 2 Sharett Institute of Oncology, Hebrew University-Hadassah Medical Center, 9103401 Jerusalem, Israel; 3 Department of Developmental Biology and Cancer Research, Institute for Medical Research-Israel-Canada, The Hebrew University-Hadassah Medical School, 91120 Jerusalem, Israel; 4 The Legacy Heritage Oncology Center & Dr. Larry Norton Institute, Soroka University Medical Center, Ben Gurion University of the Negev, Faculty of Medicine, 8410101 Beer Sheva, Israel; 5 School of Software Engineering and Computer Science, Azrieli College of Engineering, 9103501 Jerusalem, Israel

**Keywords:** Cancer resistance, Intra-tumor heterogeneity, Tumor plasticity, Information-theoretic single-cell analysis, Individualized targeted therapy, Radiation oncology, Triple-negative breast cancer

## Abstract

**Background:**

Drug resistance continues to be a major limiting factor across diverse anti-cancer therapies. Contributing to the complexity of this challenge is cancer plasticity, in which one cancer subtype switches to another in response to treatment, for example, triple-negative breast cancer (TNBC) to Her2-positive breast cancer. For optimal treatment outcomes, accurate tumor diagnosis and subsequent therapeutic decisions are vital. This study assessed a novel approach to characterize treatment-induced evolutionary changes of distinct tumor cell subpopulations to identify and therapeutically exploit anticancer drug resistance.

**Methods:**

In this research, an information-theoretic single-cell quantification strategy was developed to provide a high-resolution and individualized assessment of tumor composition for a customized treatment approach.

Briefly, this single-cell quantification strategy computes cell barcodes based on at least 100,000 tumor cells from each experiment and reveals a cell-specific signaling signature (CSSS) composed of a set of ongoing processes in each cell.

**Results:**

Using these CSSS-based barcodes, distinct subpopulations evolving within the tumor in response to an outside influence, like anticancer treatments, were revealed and mapped. Barcodes were further applied to assign targeted drug combinations to each individual tumor to optimize tumor response to therapy.

The strategy was validated using TNBC models and patient-derived tumors known to switch phenotypes in response to radiotherapy (RT).

**Conclusions:**

We show that a barcode-guided targeted drug cocktail significantly enhances tumor response to RT and prevents regrowth of once-resistant tumors. The strategy presented herein shows promise in preventing cancer treatment resistance, with significant applicability in clinical use.

**Supplementary Information:**

The online version contains supplementary material available at 10.1186/s13073-022-01121-y.

## Background

Drug resistance and cancer cell plasticity are principal contributors to therapeutic failure [1, 2]. Discovering a strategy with the ability to transform the potential evolution of certain intra-tumor subpopulations within treated/irradiated tumors into a therapeutic advantage, is an unmet need in cancer research and clinical practice [2].

We propose a novel approach where cancer treatment can be designed based on the changes occurring in patient-specific intra-tumor subpopulations in response to radiotherapy (RT) or cytotoxic treatment. In this study, this approach was assessed using a triple-negative breast cancer (TNBC) model and patient-derived TNBC samples treated with RT.

Recent studies have shown that although being an established and effective anti-cancer treatment, radiation may promote anti-apoptotic and pro-proliferative responses that often result in tumor regrowth [3, 4]. This has initiated numerous attempts to characterize tumor molecular phenotypes expressed in response to RT to identify new potential drug targets and strategies for anti-cancer treatment enhancement [5–10].

TNBC is a clinically unique, aggressive, and highly heterogeneous subtype of breast cancer that does not express estrogen receptors, progesterone receptors, or human epidermal growth factor receptor-2 (Her2), and for which no targeted therapy exists. Chemotherapy (CT) and RT have remained the standardized treatment options for the past 20 years [11, 12]. While TNBC may be sensitive to RT initially, resistance often develops at later stages [12] due to significant intra-tumor cell heterogeneity [13, 14] and potential phenotypic switching due to cellular plasticity (e.g., from Her2^-^ to Her2^+^) [15].

This study proposes an information theoretic approach utilizing thermodynamic-based surprisal analysis (SA) [16] in single cells to elucidate TNBC cellular subpopulations at a high resolution. We quantify evolving subpopulations in response to RT through single-cell barcoding of ongoing processes in TNBC tumor cells. Thermodynamic-based information theory is implemented to identify ongoing processes within each cell. Tumors may be considered as homeostatically disturbed entities that have deviated from a balanced state due to various constraints (e.g., mutational stress and drug treatment) [16]. Each constraint creates a deviation in the expression levels of a subset of proteins in the tumor. In doing so, an ongoing (unbalanced) process in the tumor, consisting of the group of proteins that were altered by the constraint, becomes active. SA identifies the constraints operating in a system as well as the proteins affected by each constraint.

We have previously demonstrated that accurate identification of unbalanced processes in human cells using bulk SA can anticipate the effect of protein inhibitors on protein co-expression network structures [17]. SA of cell-cell signaling in brain tumors has also been shown to predict cellular spatial distributions and the direction of cell-cell movement [18]. Additionally, we have applied SA to large-scale proteomic datasets for the prediction of efficient patient-specific targeted combination therapies [19–21].

In this study, SA is extended to identify cell-specific signaling signatures (CSSSs), consisting of a unique set of ongoing processes that have emerged within the individual cell. Each CSSS is converted into a cell-specific barcode. An intra-tumor subpopulation is then defined to be a group of cells harboring the exact same CSSS-based barcode and these cells are expected to respond similarly to treatment.

The final result of such an analysis is a high-resolution intra-tumoral map of the different subpopulations within a tumor and the CSSS that characterizes every subpopulation. Such a robust and comprehensive map provides guidance on the accurate determination of drug combinations to effectively target evolving subpopulations within the tumor to bring about a potent therapeutic effect.

## Methods

### Computational methods

#### Single-cell computational data analysis

**Fig. 1:**
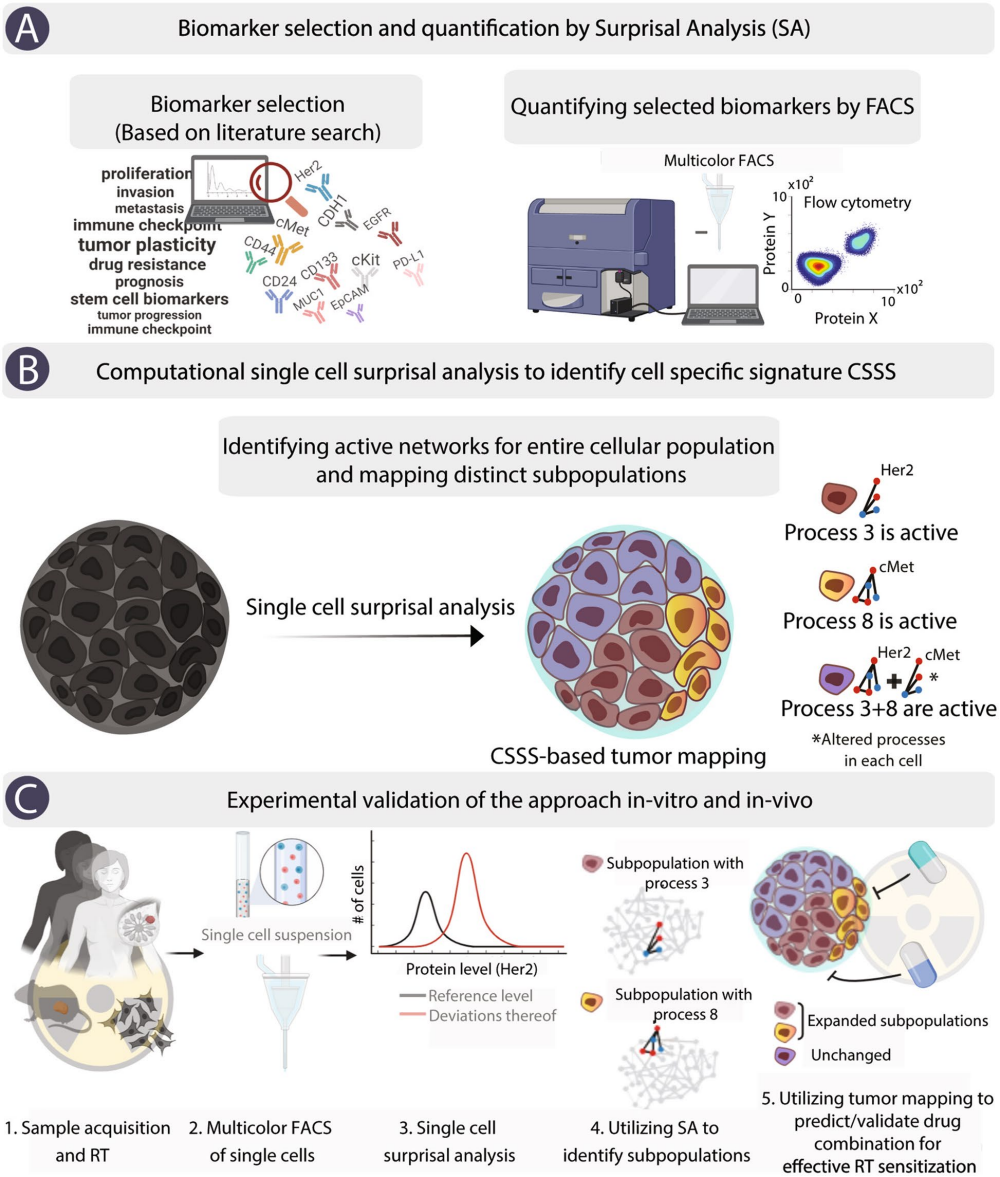
Study scheme. **A** A literature search was used to compose a list of oncomarkers for single-cell quantification (left panel). Selected cell-surface oncoproteins were quantified using multicolor FACS (right panel). **B** SA was extended to single cells to identify distinct subpopulations based on *sets* of unbalanced processes (cell-specific signaling signatures, CSSS, right panel). **C** To validate the hypothesis that targeting evolving cellular subpopulations in response to RT would enhance TNBC RT sensitization, a series of in vitro and in vivo experiments, using TNBC patient-derived and mice models, were performed

**Fig. 2:**
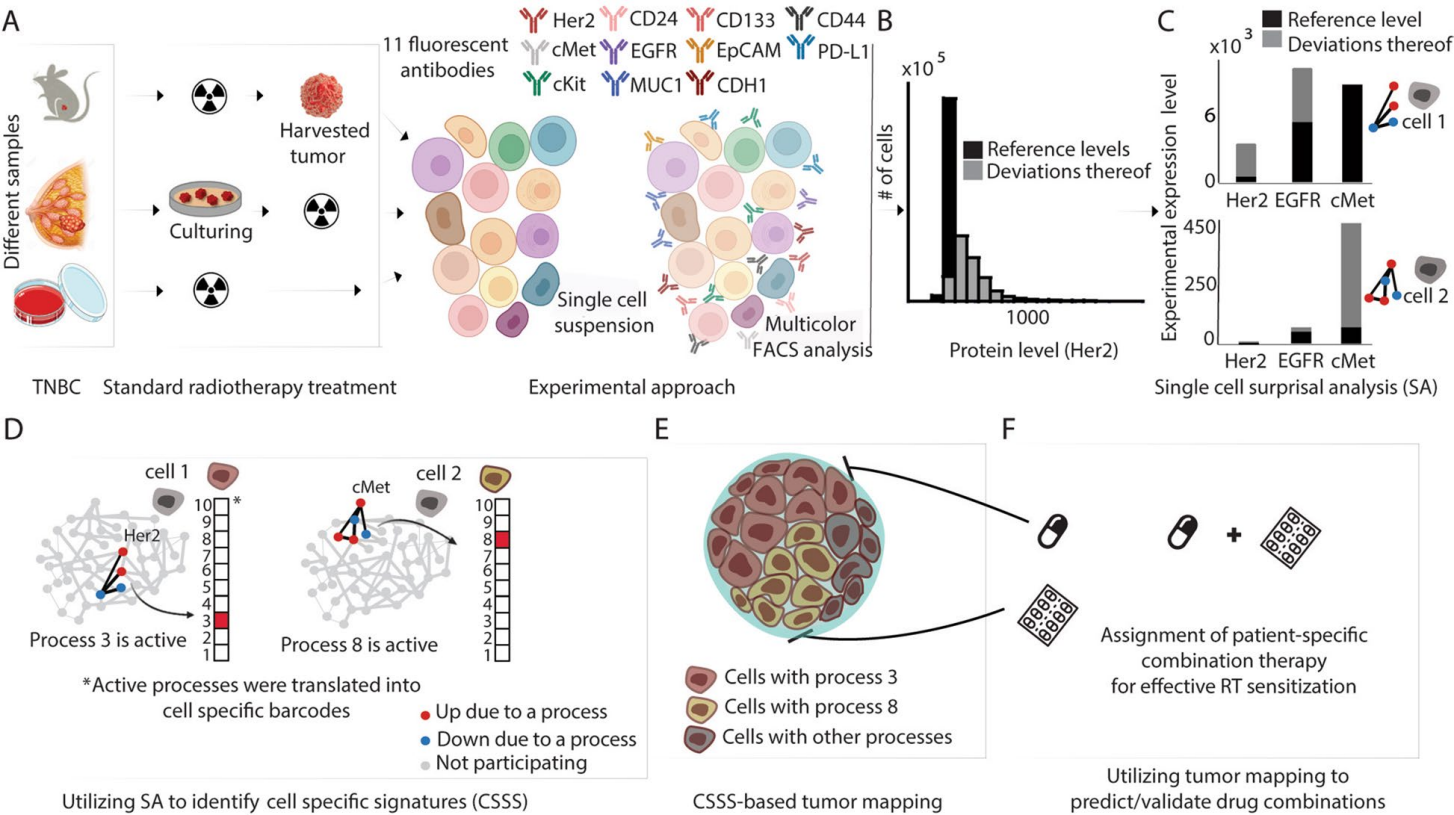
Schematic of the application of the surprisal analysis algorithm*. ***A** Preparation of fluorescently-tagged single-cell suspensions from different sample sources (control and post-RT) for multicolor FACS analysis. Each cell was labeled with a mixture of 11 fluorescently tagged antibodies. **B** Surprisal analysis reveals protein expression level distributions at the reference (steady) state and the deviations thereof due to constraints in the system (e.g., irradiation). An example for calculated distribution of the expression levels at the reference state and deviations thereof is presented for Her2, initially quantified by FACS and analyzed by SA, in 4T1 mice model of TNBC. **C** Proteins deviating from the steady state in a coordinated manner are grouped into altered subnetworks referred to as “unbalanced processes.” For example, in one 4T1 cell, the levels of Her2 and EGFR deviate significantly (upregulated) from the steady state and in the other cells, cMet levels deviate significantly (upregulated as well). Thus, the two cells are defined by the analysis as possessing different processes. **D** The unbalanced processes in each cell provide a cell-specific signaling signature (CSSS). Each CSSS is schematically transformed into a cell-specific barcode, indicating active and inactive processes. **E** Cells sharing the same barcode are organized into distinct subpopulations. **F** Tumor-specific targeted therapy combinations are tailored against the subpopulations expanding in response to RT

Surprisal analysis (SA) was applied on the single-cell level so that each cell could be plotted according to its molecular aberrations and network reorganization (Figs. 1 and 2).

#### Surprisal analysis

SA is a thermodynamic-based information-theoretic approach [22–24] which has recently been implemented to analyze bulk [16, 19, 20, 25] and single-cell biological data [25, 26]. The analysis is based on the premise that biological systems reach a balanced state when the system is free of constraints [27–29]. However, when under the influence of environmental (e.g., exposure to a drug) and/or genomic constraints (genomic mutations that affect transcript/protein expression and function), the system is prevented from reaching a state of minimal free energy, and instead reaches a state of higher free energy—a constrained state [16].

Expression levels of various macromolecules, e.g., transcripts or proteins are used as an input for SA. Since the varying constraints that act upon living cells ultimately manifest as alterations in the cellular protein/gene expression network, they are viewed as emerging *unbalanced molecular processes* [19, 30]. Recent examples of SA implementation in biology include the characterization of bulk proteomic changes in large datasets, including multiple patient tissues and cancer cell lines, to predict a change in the behavior of systems [17, 18] or to design individualized drug therapies [19–21].

In this study, we analyzed protein expression data obtained from multicolor FACS in which each cell was labeled with a mixture of 11 fluorescently tagged antibodies. Additional file 1 provides the details for the models and single-cell analyses used in the study. It is important to note that this methodology may be applied to any single-cell proteomics data. The data matrix obtained from the flow cytometry analysis (~100,000–500,000 cells), in which columns are protein expression levels and rows are single cells, was used as an input for surprisal analysis. RT treatment imposes a constraint, but more than one constraint may be identified in the system.

Equation 1 was used to identify different constraints within tumor cells:

1 $$\underbrace{X_i(cell)}_{\begin{array}{c}\mathrm{experimental}\\\mathrm{level}\;\mathrm{of}\;\mathrm{protein}\;i\end{array}}=\underbrace{X_i^o(cell)}_{\begin{array}{c}\mathrm{level}\;\mathrm{of}\;\mathrm{protein}\;i\\\mathrm{in}\;\mathrm{the}\;\mathrm{reference}\;\mathrm{state}\end{array}}\exp\underbrace{\left(-\sum\nolimits_{\alpha=1}G_{i\alpha}\lambda_\alpha(cell)\right)}_{\begin{array}{c}\mathrm{changes}\;\mathrm{in}\;\mathrm{protein}\;\mathrm{levels}\\\mathrm{due}\;\mathrm{to}\;\mathrm{the}\;\mathrm{constraints}\;\alpha=1,2,..\end{array}}$$

**Fig. 3:**
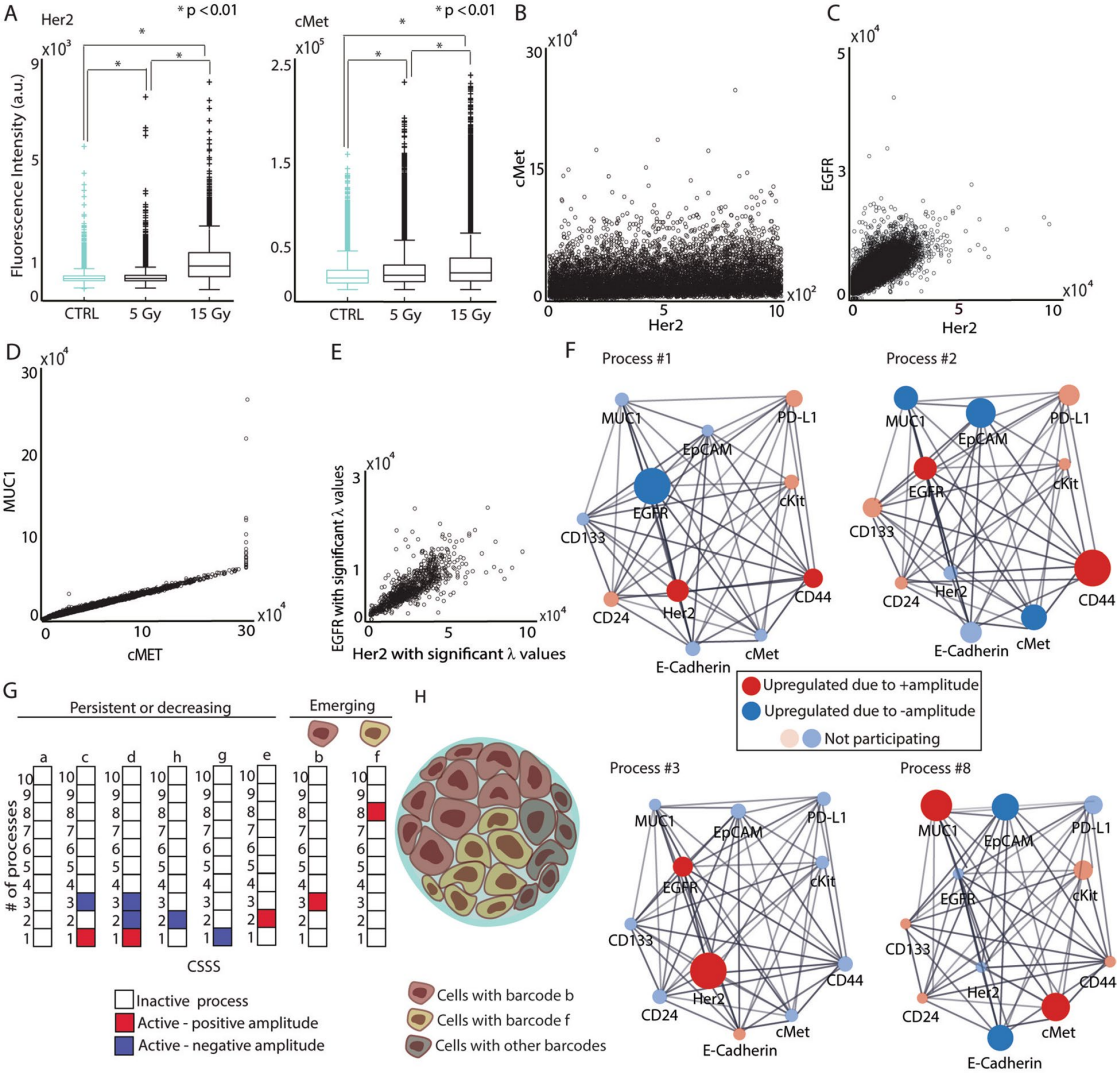
Resolving expanded 4T1 cellular subpopulations post-RT. **A** FACS expression levels of Her2 and cMet following RT. **B–D** Correlation plots between Her2 and cMet (**B**), Her2 and EGFR (**C**), and MUC1 and cMet (**D**) in irradiated cells. **E** Correlation plot between Her2 and EGFR levels expressed in the cells found to harbor process 3 (only cells with significant amplitudes (*λ*_3_(*cell*) values) were included in this plot, also see the “Methods” section). **F** Protein–protein networks were generated using single-cell SA analysis and STRING to assign the functional connections. To determine the direction of change in every protein (i.e., upregulation or downregulation) a sign of the amplitude in a process *α* in each cell was considered. Four unbalanced subnetworks (processes) out of 10 resolved in 4T1 (Additional file 3) are shown. **G** Each cell was assigned a barcode representing a cell-specific signature (CSSS). The most abundant (>1%) subpopulations are presented. **H** Based on these CSSSs the tumor was divided into distinct subpopulations. Quantification of subpopulations was performed using at least ~30,000 cells from each condition, which were obtained from at least three flasks and from at least three independent experiments for each time point. For **A:** statistically significant differences between control and 5 Gy; control and 15 Gy; and 5 Gy and 15 Gy were determined using a two-tailed Student’s *t* test (*P < 0.01)

Here, ${X}_i^o(cell)$ is the expected expression level of protein *i* at the reference state in a measured cell. The exponential term in Eq. (1) represents the deviation from the reference value due to the constraints, including those imposed by irradiation. *G*_*iα*_ are weights (the degree of participation) of a protein *i* in the unbalanced processes *α* = 1, 2... Proteins deviating in a similar manner from the steady state are grouped into unbalanced processes (see examples for 2 cells quantified in the 4T1 TNBC model in Fig. 2C). *λ*_*α*_(*cell*) is the amplitude of an unbalanced process *α* = 1, 2. . in each tested cell. For example, Additional file 2: Fig. S2 presents *λ*_*α*_(*cell*) values for process 8 (the network representing process 8 is shown in Fig. 3F and generated as explained below) in untreated and irradiated cells (6 days post-RT) in the 4T1 in-vitro model. *Negative/positive* amplitude denotes how the cells are correlated with respect to a particular process. The processes are indexed *α* = 1, 2, 3… so that the significance of the process decreases with an increasing index, i.e., unbalanced process 1 appears in a higher number of cells than unbalanced processes 2, 3, etc. *Several* unbalanced processes may be found in a system, however not all processes are active in all cells (see in the sections below how we define whether a particular process is active in a tested cell). ∑_*α* = 1_*G*_*iα*_*λ*_*α*_(*cell*) represents the amount of information we have about each protein *i*. The partial deviations in the expression level of protein *i* due to the different constraints add up to the total change in expression level. A protein that is influenced by constraints, i.e., is influenced by one or more unbalanced processes, cannot take on any possible expression level. Its expression level is affected by the expression levels of other proteins in the unbalanced process in the cell.

##### Calculation of *λ*_*α*_(*cell*) and *G*_*iα*_

We fit the main Eq. (1), to the logarithm of the measured expression level of protein *i* in each cell using singular value decomposition (SVD). *In practical terms*, a matrix is constructed, containing the natural logarithm of protein expression levels in the different cells [26]. The procedure then utilizes SVD as an intermediate step, which calls for the construction of two square (and symmetric) co-variance matrices. One is smaller with a maximal rank of 11 (as the number of the proteins) and the second is bigger (depends on the number of cells, in this case at least 100,000). These matrices are diagonalized to calculate eigenvectors and eigenvalues. SVD and all other mathematical calculations described here were implemented using Matlab. Codes can be found in Github [31]. Eigenvectors and eigenvalues are further used by SA to calculate the amplitudes of the processes: *λ*_*α*_(*cell*) for each cell if we use single-cell data, *λ*_*α*_(*k*) for each sample *k* if we use bulk data [19, 20, 30], and *G*_*iα*_ values, which are weights of the proteins in each process *α*. A detailed, step-by-step description of the mathematical procedure, namely how the eigenvectors and eigenvalues are used to calculate the amplitudes (*λ*_*α*_(*cell*) or *λ*_*α*_(*k*)) and *G*_*iα*_ values, is described in the Supplementary Information of reference [16]). Any additional information regarding the mathematical procedures/codes can be provided upon request.

The number of calculated constraints is limited by the smaller dimension of the input matrix. In this case, it was limited to 11 (the number of measured proteins) and therefore a maximum of 10 constraints or unbalanced processes (10 constraints plus steady state) could be found. Calculations of the parameters using a smaller matrix (detailed in [16]) allow for the fast and efficient data processing of hundreds of thousands of cells. The number of proteins quantified in each cell can be significantly expanded (hundreds or thousands) without significantly increasing the data processing time.

##### Generation of protein-protein networks representing unbalanced processes

Additional file 2: Fig. S1 complements Fig. 3F and shows additional unbalanced processes active in the 4T1 system. The goal was to generate unbalanced processes composed of proteins with significant *G*_*iα*_ values. *G*_*iα*_ sign indicates the *correlation or anti-correlation* between proteins in the same process (Additional file 3). Upregulation or downregulation due to process *α* can be defined further using a product *G*_*iα*_*λ*_*α*_(*cell*) for each protein in every *cell* in each experimental condition/time point: e.g., proteins with positive *λ*_*α*_(*cell*) and positive *G*_*iα*_ will be upregulated due to process *α*, since the product *G*_*iα*_*λ*_*α*_(*cell*), which represents a deviation from the steady state due to process *α*, will be positive. Protein-protein interactions in each unbalanced process are based on the STRING database [32]. The radius of each circle in the map corresponds to the *G*_*iα*_ value (Fig. 3F).

##### Calculation of cell-specific barcodes based on CSSS

It is important to note that not all processes are active in all cells. The term *λ*_*α*_(*cell*) represents the importance of the unbalanced process *α* in the *cell*. Its sign indicates the correlation or anti-correlation between the same processes in different cells. To further map distinct subpopulations within the entire cellular population, we grouped cells sharing *the same set* of unbalanced processes, indicated by the cell-specific signaling signatures (CSSS), into distinct subpopulations (Figs. 1 and 2). Only unbalanced processes with significant amplitudes were included in the CSSS of each individual cell as follows:

To determine threshold limits for *λ*_*α*_(*cell*) values, *λ*_*α*_(*cell*) values were sorted and plotted as sigmoid plots in each process. Only *λ*_*α*_(*cell*) values located on the tails of the sorted distributions were considered and used further for the barcode calculations (Additional file 2: Fig. S2).

**Fig. 4:**
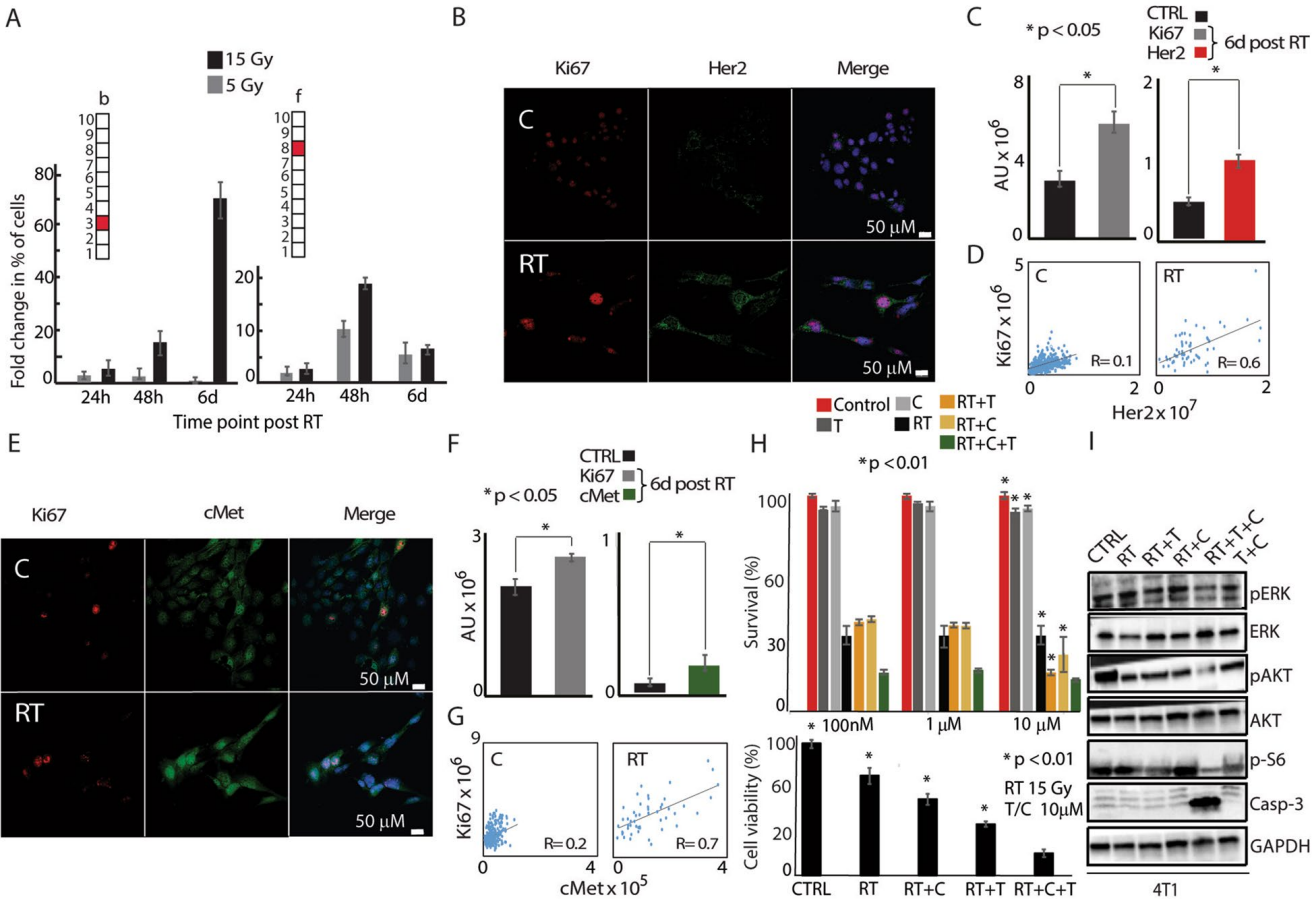
Two distinct subpopulations expand and show proliferative properties in response to RT. **A** Very small subpopulations (<1%), represented by barcodes b and f, expanded significantly following RT (fold change in % of cells relative to the control of each time point). **B–G** 4T1 cells were irradiated with 15Gy. 6 days post-RT, cells were incubated with antibodies against Ki67, cMet, and Her2 and nuclei were stained with DAPI (fluorogel II). **B**, **E** 40× lens; scale bar represents 50 μm. **C**, **F** Sum intensities of Ki67 (**C**, left panel); Her2 (**C**, right panel); Ki67 (**F**, left panel); and cMet (**F**, right panel) were calculated from 8 to 10 fields using the NIS-Elements software (Nikon). **D**, **G** Correlation plots between **D** Ki67 and Her2 and **G** Ki67 and cMet were generated for each indicated condition to test co-activation represented in **C**, **F**. *R* values indicating the degree of correlation between Ki67 and Her2 (**D**) and Ki67 and cMet (**G**) were calculated before and after RT. **H** Survival rates of 4T1 cells in response to Trastuzumab (T), Crizotinib (C), RT, RT+C, RT+T, and RT+T+C as detected by MB survival assays 6 days post RT (upper panel), and cell viability as measured by the MTT assay (lower panel). Drugs were added from 3 days prior to RT until the end of the experiment. **I** Key downstream to Her2 and cMet signaling proteins are shown following different treatments. The predicted combination induced high levels of cleaved caspase-3 compared to radiation alone, irradiation+T, and irradiation+C. Downregulation of pAKT, pERK, and p-S6 was detected when T+C was applied prior to RT. For **A**: quantification of subpopulations was performed using at least ~30,000 cells from each condition, which were obtained from at least three flasks and from at least three independent experiments for each time point. For **C** and **F**, statistically significant differences between all presented conditions and the cells treated with RT+T+C were determined using a two-tailed Student’s *t* test (*P < 0.05); for **H**, upper and lower panels *P < 0.01

The combinations of unbalanced processes (CSSS) for each cell were generated using *λ*_*α*_(*cell*) values that exceeded threshold limits. In this way, CSSSs were assigned to each cell and were converted into cell-specific barcodes for simple representation. Additional file 3-Additional file 8 include the input FACS data and the output parameters obtained using CSSS analysis (*λ*_*α*_(*cell*) and *G*_*iα*_, and barcodes denoting subpopulations) for the major cancer systems, 4T1 and BR45, which have been tested in vitro and in vivo. Based on these cell-specific barcodes, distinct subpopulations were determined in the tumor (Fig. 3G, H). Subpopulations *b* and *f* (Fig. 4A) expanded significantly as detailed in the main text.

##### CSSS vs PCA and tSNE

Several dimensionality reduction algorithms have been developed to interpret single-cell variations (e.g., variations in protein or gene expression levels), such as clustering-based t-SNE analysis [33] or principal component analysis (PCA) [34–36]. These methods are very useful in the statistical determination of dominant expression patterns but are limited when a more deterministic partitioning of a tumor mass into cellular subpopulations, based on cell-specific sets of altered molecular processes, is required. For example, t-SNE is a non-deterministic method (e.g., different runs with the same hyperparameters may produce different results) and is unable to assign a certain protein to several processes, or to determine which processes are active in every cell (Additional file 2: Fig. S3). Therefore t-SNE will be less efficient when the determination of robust cell-specific signaling signatures is required (e.g., for drug combination design). Similarly, PCA focuses mainly on the most dominant patterns obtained from proteins with the highest variability in the population, rather than on cell-specific sets of altered processes [30, 37]. Additional file 2: Fig. S3 and Fig. S4 show separation of the 4T1 single-cell data, obtained using either t-SNE or PCA analysis (performed using Python), using 2 main principal components. Minority separation between control and RT-treated cells and within RT-treated cells can be observed and therefore CSSS analysis was vital in identifying the two separate subpopulations, b and f, that expanded in response to RT, and were mapped and quantified (Additional file 2: Fig. S3 and Fig. S4, see also Figs. 3-4 and main text for more details).

### Experimental methods

#### Patient-derived tissue used to establish BR45 tumors

Patient-derived tumors were established from a female patient with triple-negative, invasive lobular breast cancer. The tissue was derived from the local chest wall recurrence, s\a mastectomy, chemotherapy, and radiotherapy. When implanted into the NSG mice the tissue formed a tumor, and then was used for the in-vivo experiments as described below.

#### Cell lines

Murine 4T1 cells were kindly provided by Dr. Zvika Granot. MDA-MB-468 and MDA-MB-231 cells were acquired from ATCC and authenticated by the Genomic Center of the Technion Institute (Haifa). BR45 PDX were obtained from the Oncology Department at Hadassah –Jerusalem Medical Center with prior written informed consent. The BR45-derived and 4T1 cells were maintained and irradiated, after which flow cytometry was performed as indicated in Additional file 2: Supplementary Information file (SI).

#### Murine models

##### Syngeneic model

2.0×10^5^ 4T1 cells were inoculated subcutaneously into 6–7-week-old female BALB/c mice (ENVIGO).

##### Allogeneic model

BR45 tumors were induced in NSG (Jackson Laboratory) female mice either by injecting 4.0×10^6^ cells or by transplanting xenografts, orthotopically.

After reaching the initial volume 80–100 mm^3^, mice were randomly grouped to approximately 8–10 animals per cage, and treatment was initialized. Tumor sizes were routinely measured with an electronic caliper every two days and their volumes were obtained using the formula V = (W (2) × L)/2. All in vivo experiments were performed with the approval of the Hebrew University of Jerusalem IACUC. See Additional file 2: SI Methods for more details.

### In vivo treatments

High dose rate (HDR) brachytherapy (GammaMed™ HDR, Iridium 192) was performed as previously described [38]. 12 Gy was administered on two alternative days. Trastuzumab was purchased from Teva Pharmaceutical Industries Ltd. Crizotinib (cMet inhibitor, #12087-50) and Erlotinib (EGFR inhibitor, #10483-1) were purchased from Cayman Chemical. (See Additional file 2: SI Methods for doses and regimens).

#### Cohort description for each type of in vivo experiment performed

##### 4T1 In vivo experiment

First experiment: 4T1 cells (2 ×10^5^) were subcutaneously injected in female BALB/c mice.

Mice that reached a tumor volume of 80–100 mm^3^ were divided into two groups: control and irradiated. The irradiated group was treated by brachytherapy on two alternate days (12 Gy/day). Each group had two exit points: 6 and 12 days. Control samples *n*=12 (5 mice at day 6 post-RT and 7 mice at day 12 post-RT); RT-treated samples *n*= 17 (9 mice at day 6 post-RT and 8 mice at day 12 post-RT).

At the end of each time point mice were terminally anesthetized with Ketamine-Xylazine (150 mg/kg/20 mg/kg) IP, after which mice were euthanized by cervical dislocation. An incision was made and the tumor mass as a whole was gently separated from the conjunctive tissue using a sharp blade. Six and 12 days post-RT, whole tumors were collected for FACS analysis after mechanical cell dissociation. CSSS analysis was performed using FACS output.

Second experiment: The setup and procedure of collecting and analyzing the output was exactly as described in the first experiment. When tumors reached volumes of 80–100 mm^3^, mice were divided into four groups: each group had two exit points — 6 and 12 days. Mice were treated with 5 mg/kg Trastuzumab (T) and 25 mg/kg Crizotinib (C) starting 3 days prior to brachytherapy until the end of the experiment (day 17). Control: *n*=12 (7 mice at day 6 post-RT and 5 mice at day 12 post-RT), RT: *n*= 11 (5 mice at day 6 post-RT and 6 mice at day 12 post-RT), RT+T+C: *n*= 7 (4 mice at day 6 post-RT and 3 mice at day 12 post-RT), T+C: *n*= 10 (5 mice at day 6 post-RT and 5 mice at day 12 post-RT), RT+T: *n*= 12 (6 mice at day 6 post-RT and 6 mice at day 12 post-RT). For more details see Additional file 1.

##### BR45 PDX in vivo experiments

A small portion (~30 mm^3^) of BR45 PDX was transplanted orthotopically into each NSG female mouse. When tumor volumes reached 80–100 mm^3^, mice were divided into seven groups; each group had two exit points: 6 and 12 days. Mice were treated with 5 mg/kg Trastuzumab (T), 25 mg/kg Crizotinib (C), and 12.5 mg/kg Erlotinib (E) starting 3 days prior to RT until the end of the experiment (day 17). Control: *n*=6 (3 mice at day 6 post-RT and 3 mice at day 12 post-RT), RT: *n*= 5 (3 mice at day 6 post-RT and 3 mice at day 12 post-RT), RT+T: *n*= 6 (3 mice at day 6 post-RT and 3 mice at day 12 post-RT), RT+C: *n*= 6 (3 mice at day 6 post-RT and 3 mice at day 12 post-RT), RT+T+C: *n*=6 (3 mice at day 6 post-RT and 3 mice at day 12 post-RT), RT+T+C+E: *n*= 6 (3 mice at day 6 post-RT and 3 mice at day 12 post-RT), T+C: *n*= 7 (4 mice at day 6 post-RT and 3 mice at day 12 post-RT). Mice were irradiated by brachytherapy on two alternate days with two doses (12 Gy and 10 Gy). The setup procedure of collecting and analyzing the output data was exactly as described in the 4T1 model. For more details, see Additional file 1.

##### Flow cytometry

Each sample was labeled with an 11 fluorescently tagged antibody (Ab) mixture. In addition when analyzing tumors, an exclusion cocktail including anti-mouse CD45, CD31 and CD140 was used in *in-vivo* experiments to exclude adjacent stromal and immune cells (Additional file 2: Table S8). A LSR-Fortessa Analyzer was utilized to measure all biomarkers simultaneously in each cell. See Additional file 2: SI Methods for more details.

##### Western blot analysis

Cell pellets were lysed with a 20% SDS buffer (targeted drugs were added 1 day prior to RT, which allowed to obtain enough protein concentration for WB). The protein content of each lysate was determined with a Pierce BCA Protein Assay Kit (#23225, ThermoFisher). Equal protein aliquots were subjected to SDS-PAGE (Criterion Stain Free, 4–15% acrylamide, Bio-Rad) under reducing conditions and proteins were transferred to a nitrocellulose membrane. (Millipore). Membranes were blocked with 5% non-fat milk for 1 hour at R.T. and probed with the appropriate antibody (Additional file 2: SI Methods), followed by horseradish peroxidase-conjugated secondary antibody (#123449, Jackson ImmunoResearch) and a chemiluminescent substrate (ECL #170-5061, Bio-Rad).

##### Survival assay

Cells were seeded at 70% confluency and treated as required for different time points. Cells were washed with PBS and fixed with 4% PFA for 10 min at R.T. The fixed cells were stained with methylene blue (MB) for 1 hour at R.T., washed and air dried overnight. The dye was extracted with 0.1M HCl for 1 hour at R.T. Absorbance was read at 630 nm.

##### MTT assay

Cells were seeded and treated as indicated in a 96-well plate for 6 days. Cell viability was determined using an MTT assay kit (#ab211091, Abcam). Equal volumes of MTT solution and culture media were added to each well and incubated for 3 h at 37 °C. MTT solvent was added to each well, and the plate was then covered with aluminum foil and put on the orbital shaker for 15 min. Absorbance was read at 590 nm after 1 h.

##### Immunofluorescence

Cells were grown on coverslips in six-well plates to reach 70% confluency by the next day, then fixed and permeabilized with cold absolute methanol. Afterwards, they were blocked with CAS blocker (cat. no. ZY-008120) and washed 3 times for 5 min with PBS, then stained with the following primary antibodies: Anti-mouse/human Ki-67 (BLG-151202), Rabbit Anti-Met (cMet) Polyclonal Antibody (BS-0668R), Neu (F-11) SC-7301. After washing 3 times with PBS for 5 min, cells were stained with secondary antibodies for 1 h at room temperature in the dark to visualize the aforementioned primary antibodies. The secondary antibodies conjugated to fluorophores were as follows: Goat anti-rat IgG H&L conjugated with Alexa Fluor 647 (1:400) (cat. no. 712605153), Goat anti-mouse IgG (H+L) conjugated with Alexa Fluor 488 (1:150) (cat. no. 115545003), and Goat anti-Rabbit IgG (H+ L) conjugated with Alexa Fluor 488 (1:150) (cat. no. 111545003). All secondary antibodies were purchased from Jackson ImmunoResearch. After washing 3 times with PBS, cell slides were mounted using fluorogel III mixed with DAPI (EMS, cat. no. 17985-01) to stain the nuclei. A spinning disk confocal microscope was used to visualize the expression of biomarkers of interest. The analysis was done using NIS-Elements software (Nikon).

##### Experimental statistical analysis

Significant differences between experimental conditions and experimental reproducibility were determined using the Student’s *t*-test (two tails, two samples equal variance); *P* values of ≤0.05 were considered statistically significant. All data was represented as the mean ± S.E. (standard error of the mean) if not indicated otherwise. Quantification of subpopulations was performed using a minimum of 30,000 cells from each condition, which were obtained from at least three flasks and from at least three independent experiments for each time point/condition. All experiments were performed minimally in biological triplicate if not indicated otherwise.

##### Code availability statement

All equations and mathematical procedures used in this article are detailed in the “Methods” section and/or referenced. The approach is covered by patent applications “A method for selecting patient specific therapy”, PCT/IL2019/050474, and “Methods of Determining Cancer Therapy,” PCT/IB2021/056136. Any additional clarification/information regarding mathematical procedures/codes can be provided upon request. The codes for single-cell computational analysis are publicly available from Github [31].

## Results

### Study overview

To collect high-resolution data regarding the intra-tumoral composition of TNBC tumors in response to RT, we employed the following computational-experimental strategy: (1) A list of cell-surface oncomarkers for single-cell quantification and analysis (Methods) was determined using a literature search (Fig. 1A, left panel). (2) Single-cell suspensions from multiple sources (e.g., cell lines, tumors from mice, or patient-derived models) were labeled with fluorescently-labeled antibodies targeting selected cell-surface oncoproteins and assayed by multicolor FACS to reveal oncoprotein expression levels in each cell (Fig. 1A, right panel). In every experimental condition, 30,000–50,000 single cells were profiled allowing for the identification of different subpopulations, including very small subpopulations (comprising less than 1% of the total population) that have significantly limited detection rates when using standard pathological tests. (3) SA was extended to single cells (“Methods”) to identify *sets* of unbalanced processes (cell-specific signaling signatures (CSSS)) in each cell, thereby identifying distinct cellular subtypes within the tumor (Fig. 1B). (4) We hypothesized that targeting evolving cellular subpopulations in response to RT would enhance TNBC response to RT and inhibit RT resistance development. A series of in vitro and in vivo experiments were performed to validate this hypothesis (Fig. 1C) as detailed in the sections below (Additional file 1 includes the metadata describing all the TNBC models used in the study).

### CSSS analysis

We selected 11 cell surface proteins for single-cell quantification using an extensive literature search on relevant oncomarkers for breast cancer [39–45]. The list of oncomarkers included Her2, EGFR, EpCAM, CD44, CD24, PD-L1, cKit, CD133, E-Cadherin, cMet and MUC1.

These oncomarkers are known to be involved in breast cancer proliferation with an aggressive phenotype (EGFR, Her2, MUC1,cMet, and cKit) [46–51], cancer metastasis and invasion (EpCAM, E-Cadherin, CD133, MUC1,cMet and cKit) [50, 52–55], stem cell properties (CD44, CD24, CD133) [54, 56] and immune response (PD-L1) [45], and also represent potential drug targets for breast cancer therapy (Her2, cMet, EGFR, MUC1, cKit, PD-L1).

Protein expression levels of the surface oncomarkers were quantified in single cells (Fig. 2A) using multicolor FACS and analyzed via single-cell SA (“Methods”). Single-cell protein expression levels were used to compute the steady state and deviations thereof due to unbalanced processes operating in the tumor. Proteins that deviated from the steady state (Fig. 2B) in a similar manner were grouped into unbalanced processes (Fig. 2C, “Methods”). Importantly, not all the processes identified by the analysis were active in each cell. Only processes with significant amplitudes were assigned to a cell to identify a *set* of cell-specific processes, which we termed “cell-specific signaling signature” (CSSS) (Fig. 2D). For simplicity of representation, each CSSS was converted into a cell-specific barcode, graphically representing a set of active processes in a cell (Fig. 2D, active processes are labeled in red). Based on matching CSSS we identified distinct subpopulations (Fig. 2E, “Methods”).

We suggest that the CSSS is what governs the optimal therapeutic strategy. The in-depth data collected up to this point was utilized to devise a therapeutic strategy that incorporated targeted therapies to aid RT. This was achieved by targeting dominant and RT-resistant subpopulations, to potentially achieve long-term tumor remission (Fig. 2F).

### 10 unbalanced processes give rise to the expression variations of 11 cell-surface proteins in 4T1 mouse TNBC cells

The first TNBC model used in the study included 4T1 cells, originally derived from a spontaneously arising mammary tumor in BALB/c mice and representing a model for stage IV TNBC [57]. The cells were irradiated at 5 Gy or 15 Gy and grown under standard conditions for 24h, 48h, and 6 days. Overall distribution of Her2 and cMet expression levels (Fig. 3A), as well as additional oncomarkers (Additional file 2: Fig. S5), were altered in response to RT. Two-dimensional correlation plots showed that although certain proteins, such as cMet and Her2, were upregulated in response to RT (Fig. 3A), their expression levels had poor correlation (Fig. 3B). EGFR and Her2 levels, however, demonstrated a strong correlation (Fig. 3C), as did MUC1 and cMet (Fig. 3D).

To examine all possible protein-protein relationships we utilized single-cell surprisal analysis (SA, Methods). Single-cell SA allowed for the identification and mapping of the unbalanced processes operating in the cellular population as a whole as well as in each cell.

The analysis revealed 10 unbalanced processes in the cell population. Four of the processes, all appearing in at least 1% of the treated and/or untreated cells, are shown (Fig. 3F; other processes, quantified by SA, can be found in Additional file 2: Fig. S1). Processes 3 and 8 included co-expressed and induced Her2/EGFR and cMet/MUC1 respectively (Fig. 3F, Additional file 3). Figure 3E exemplifies the procedure of mapping unbalanced processes within single cells – EGFR and Her2 show a higher correlation in the subset of cells which are assigned process 3 compared to all irradiated cells presented in Fig. 3C.

### Expansion of Her+ and cMet+ distinct subpopulations are observed in response to RT

Due to the fact that more than one unbalanced process may operate in a single cell, the sets of unbalanced processes (CSSS) in each cell were analyzed to reveal CSSS-based subpopulations. Eight dominant subpopulations, each represented by a unique barcode consisting of processes 1, 2, 3, and 8 (Fig. 3G), were found in the cell population before and/or after RT. Only CSSSs that appeared in at least 1% of the cells were considered.

When we examined the temporal behavior of the dominant subpopulations, we found that the majority of dominant subpopulations did not change or reduced in response to RT (Additional file 3). For example, subpopulation c comprised 9.9% of the cells before RT and decreased to 4.9%, 6 days post-RT. However two subpopulations b and f expanded significantly 6 days post-RT (Fig. 4A). Subpopulation b harbored only process 3, in which Her2 and to a lesser extent EGFR (Fig. 3F), were induced. Strikingly, subpopulation b was induced *~*70-fold post-irradiation relative to non-irradiated cells, where an expansion from <1% of the population in untreated cells to ~19–25% of the population 6 days post-RT occurred (Fig. 4A).

Subpopulation f harbored only process 8 (Fig. 3G), with induced cMet/MUC1 and reduced E-Cadherin. Significant induction of subpopulation f was also observed, from undetectable levels to 1.5% 6 days post-RT (Fig. 4A). These results demonstrate an important concept: although cMet and Her2 were both induced in response to RT (Fig. 3A), CSSS-based analysis revealed that these two proteins were expressed in *distinct* cellular subpopulations (processes 3 and 8 did not appear in the same cells; Fig. 3G). The development of such significant, distinct, and well-defined Her2+ and cMet+ subpopulations post-RT suggests that Her2 and cMet signaling may play a significant role in 4T1 cell survival and resistance in response to irradiation.

To characterize proliferative properties of the expanded Her2+ and cMet+ subpopulations in response to RT, we co-stained the 4T1 cell population with anti-Ki67 (proliferative biomarker), anti-cMet, and Her2 antibodies using immunofluorescent assays. Ki67, Her2, and cMet expression increased significantly in the cells surviving RT (Fig. 4B, C, E, and F). Moreover, this result was supported by enhanced coordinated expression of Her2 and Ki67 (Fig. 4D) as well as cMet and Ki67 (Fig. 4G) proteins respectively, as represented by an increased correlation between Her2 and Ki67; and cMet and Ki67 proteins, post-RT. This enhanced correlation in protein expression reveals the increased proliferative properties of Her2 or cMet expressing cells.

### Simultaneous inhibition of Her2 and cMet sensitized 4T1 TNBC model to RT treatment

We hypothesized that simultaneous inhibition of both proteins, and thus targeting of both subpopulations, may sensitize 4T1 cells to RT. Her2 and cMet represent good candidates for such a strategy, as they are both druggable oncoproteins against which FDA-approved drugs exist. To validate this hypothesis, we inhibited either each protein alone or in combination, beginning 3 days prior to RT until 6 days post-RT, after which cell survival was measured.

The Her2 inhibitor, Trastuzumab (T), and cMet inhibitor, Crizotinib (C), showed a synergistic effect in sensitizing the cells to RT (Fig. 4H, upper and lower panels). The combination of both drugs with RT increased cell death and depleted downstream signaling to Her2 and cMet, as indicated by the low levels of downstream signaling proteins pERK, pAkt, and pS6 and the enhanced cleavage of the apoptotic marker Casp3 (Fig. 4I).

**Fig. 5:**
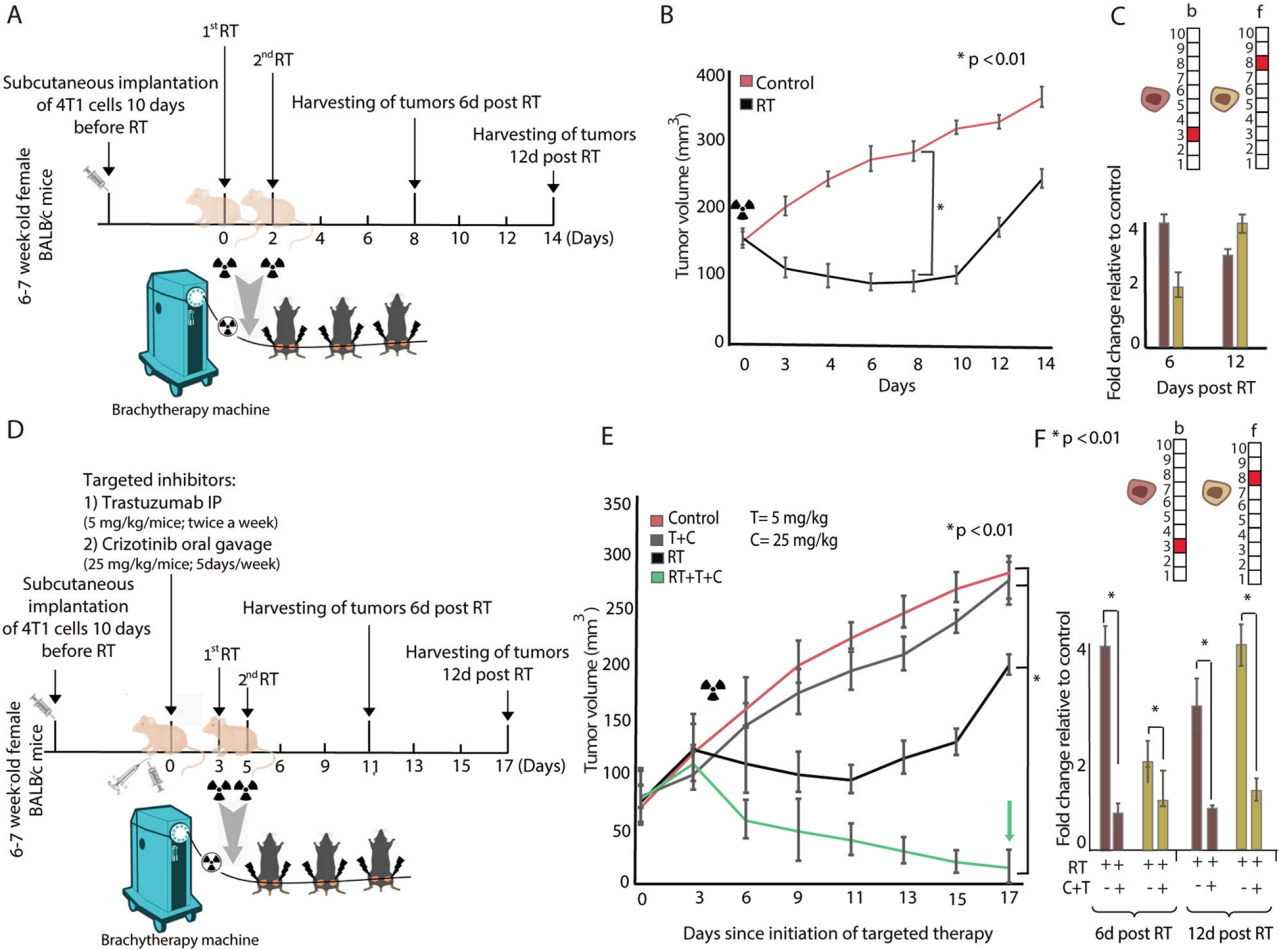
Inhibition of RT-induced subpopulations sensitized tumor response to RT. **A** 6–7-week-old BALB/c female mice were subcutaneously injected with 4T1 cells. When tumor volumes reached 80–100 mm^3^, mice were treated with brachytherapy RT on two alternate days (12 Gy). **B** Tumor volumes in the control group (*red*) versus the RT group (*black*). *p < 0.01 and ±SD are shown. **C** Fold change in the abundancy of subpopulations b and f as compared to untreated tumors. A significant expansion due to RT in subpopulation *b* harboring Her2^+^/EGFR^+^ and subpopulation f harboring cMet^+^/MUC1^+^ is detected. **D** Mice were subcutaneously injected with 4T1 cells and treated with RT. Trastuzumab (T), 5 mg/kg, and Crizotinib (C), 25 mg/kg, were administrated IP 2d/week and by gavage 5d/week respectively from d0 (3 days prior to RT) until the end of the experiment (d17). **E** C+T sensitized TNBC to RT in the 4T1 model, ± S.E., and *p < 0.01 are shown. **F** In vivo fold changes in subpopulations b and f showed optimal reduction when T and C were used in combination with RT. These results were consistent 6 and 12 days after RT. For **C** and **F**: quantification of subpopulations was performed using at least ~30,000 cells from each condition, which were obtained from at least three independent experiments for each time point, fold change is relative to control of each time point. For **B**, **C** mice used for each condition: control *n*=12, RT *n*= 11. On day 6 post-RT, we euthanized 7 control mice and 5 RT mice. On day 12 post-RT the experiment was completed and all remaining mice were euthanized. For **E**,** F** mice for each condition: control *n*= 12, RT *n*= 11, RT+T+C *n*= 7, T+C *n*= 10, RT+T *n*=12. On day 6 post-RT, 7 control mice, 5 RT mice, 4 RT+T+C mice, 6 RT+T, and 5 T+C mice were euthanized. On day 12 post-RT, the experiment was completed and all remaining mice were euthanized. For **B**, **E**, and **F**, statistically significant differences compared to cells treated with RT (**B**) and cells treated with RT+T+C (**E**, **F**) were determined using a two-tailed Student’s *t* test (*P < 0.01)

To further validate our hypothesis, we implanted 4T1 cells into immunocompetent BALB/c mice. The tumors were irradiated using brachytherapy-focused irradiation technology adapted for mice [38] by CT imaging and Monte-Carlo-based dosimetry (Fig. 5A). 4T1 tumors were then excised and single-cell suspensions were analyzed. CSSS-based analysis of the tumors 6 days post-RT, when an initial shrinkage of tumors was observed (Fig. 5B), revealed an expansion of subpopulations b and f (Fig. 5C). Moreover, 12 days post-RT, when tumor growth resumed (Fig. 5B), these expanded subpopulations were still detected (Fig. 5C, Additional file 4, Additional file 5). Inhibition of both Her2 and cMet proteins (as detailed in Fig. 5D) significantly sensitized the tumors to RT (Fig. 5E). This combined treatment resulted in tumor shrinkage and prevented the development of RT resistance (Fig. 5E). The effect of RT plus the combined targeted therapy was highly synergistic in contrast to the effect of the two targeted drugs without RT, or RT treatment alone. Furthermore, the addition of the targeted drug combination (T+C) prior to RT brought about a significant reduction in the size of subpopulations b and f (Fig. 5F). No other subpopulation expanded following the treatment (Additional file 4, Additional file 5).

### Targeting Her2 and cMet to sensitize human cell lines and patient-derived TNBC to RT

**Fig. 6:**
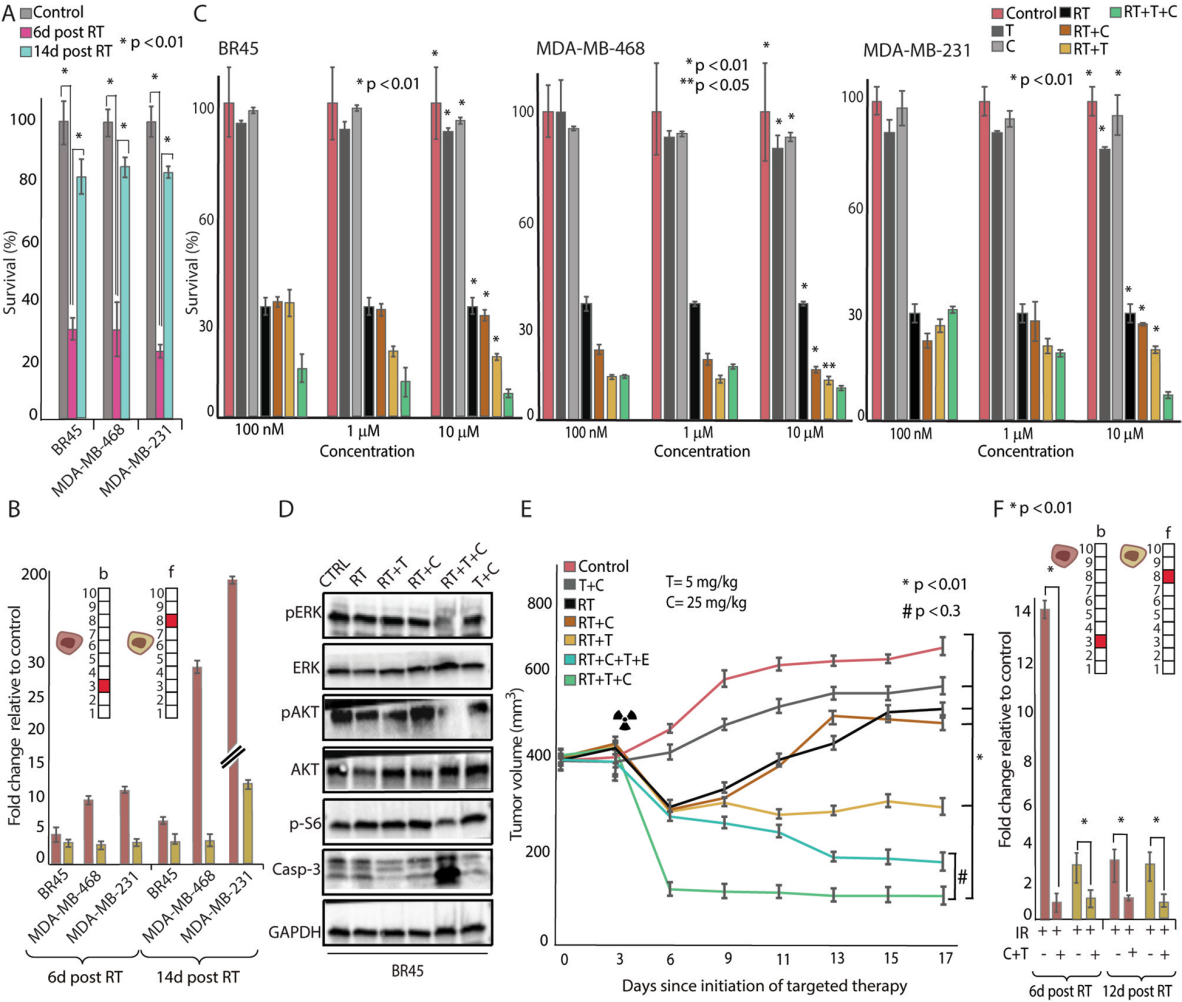
Inhibition of expanded subpopulations sensitizes human TNBC cell lines and *BR45 PDX to* RT. **A** Survival assays show a ~ 30% cell survival rate 6 days post-RT, with TNBC regrowth to ~80–90% confluency 14 days post-RT. **B** Fold changes in the abundance of subpopulations b and f compared to untreated cells. These subpopulations either remained unchanged or expanded following cellular regrowth; fold change is relative to control of each time point. **C** Survival rates of BR45, MD-468, and MD-231 cells in response to Trastuzumab (T), Crizotinib (C), RT, RT+T, RT+C, and RT+T+C 6 days post-RT. Cellular drug treatment began 3 days prior to RT and was continued until the end of the experiment (d10). **D** Downstream to Her2 and cMet signaling was tested following different treatments. C+T combined with radiation-induced higher levels of cleaved caspase-3 compared to irradiation alone and irradiation with either C or T alone or C+T. C+T administration prior to RT induced the downregulation of pAKT, pERK, and p-S6 levels. **E** C+T sensitized TNBC response to RT in BR45 PDX in vivo. BR45 tissues were transplanted orthotopically into NSG mice treated with brachytherapy on days 3 and 5 with 12 Gy and 10 Gy, respectively. Drugs were administrated from d0 (3 days prior to RT) until the end of the experiment (d17), ± S.E. are shown. **F** In vivo fold changes in the abundance of subpopulations b and f in response to T and C, which is relative to control of each time point. For **A**, **B**, **C**, and **F**, ± S.D. are shown. For **B**, quantification of subpopulations was performed using at least ~30,000 cells from each condition, which were obtained from at least three flasks and from at least three independent experiments for each time point. For **E**, **F**, mice used for each condition: control *n*=6, RT *n*= 5, RT+T *n*=6, RT+C *n*= 6, RT+T+C *n*= 6, RT+T+C+E *n*= 6, and T+C *n*=7. On day 6 post-RT, 3 control mice, 2 RT mice, 3 RT+T mice, 3 RT+C mice, 3 RT+T+C mice, 3 RT+T+C+E mice, and 4 T+C mice were euthanized. On day 12 post-RT the experiment was completed and all remaining mice were euthanized. For **A**, **C**, **E**, and **F** statistically significant differences compared to cells treated with RT (**A**) and to cells treated with RT+T+C (**C**, **E**, **F**) were determined using a two-tailed Student’s *t* test (*P < 0.01; **P < 0.05; #*P* <0.3)

To ensure that the expansion of Her2+ and cMet+ cellular subpopulations was not limited to TNBC mouse models, we tested TNBC MDA-MB-231 and MDA-MB-468 human-derived cell lines, and TNBC patient-derived cells (BR45). Inhibition of cell growth, observed in all cell types 6 days post-RT, was followed by significant cell regrowth 14 days post-RT (Fig. 6A). Subpopulations b and f, which expanded 6 days post-RT in all cell types, either maintained their size or expanded following cellular regrowth 14 days post-RT (Fig. 6B). Combined anti-Her2 and anti-cMet pretreatment sensitized all 3 types of human TNBC cells to RT (Fig. 6C). Each drug alone had a significantly smaller effect on cellular survival when compared to the combination of both drugs together with RT (Fig. 6C). Moreover, depletion of the downstream signaling pathways to Her2 and cMet as well as induction of cleaved caspase 3 were observed when the cells were pretreated with anti-Her2 and anti-cMet (Fig. 6D).

Using patient-derived TNBC BR45 cells grown in PDX models, we demonstrated that irradiated BR45 TNBC developed resistance to RT in a short period of time (tumor regrowth was detected 6 days post-RT; Fig. 6E, see black curve). Pretreatment of the mice with each drug alone prior to RT resulted in a small inhibitory effect on tumor growth (Fig. 6E). Pretreatment of the mice with the combination of both drugs, however, showed significant synergistic effects with RT, bringing about significant shrinkage of the tumor and preventing the development of resistance (Fig. 6E, green curve, Additional file 6, Additional file 7 and Additional file 8).

Adding erlotinib (an EGFR inhibitor), which according to our algorithms was not expected to significantly improve the results of the Trastuzumab + Crizotinib + RT treatment (Fig. 6E), did not significantly change the results obtained using Trastuzumab + Crizotinib + RT. Erlotinib monotherapy improved the response of BR45 to RT initially, albeit slightly, most likely due to the participation of EGFR in very small subpopulations (Additional file 7). The tumor, however, regrew after 1 week (Additional file 2: Fig. S6). Subpopulations b and f were reduced when the targeted drug combination (T+C) was applied prior to RT (Fig. 6F, Additional file 2: Fig. S7). These results suggest that CSSS-based single-cell resolution of the plasticity of TNBC in response to RT provides guidance on how effective targeted drug combinations should be designed in order to overcome RT resistance.

## Discussion

Integration of computational and biological knowledge into efficient cancer treatment design has become an emerging concept in recent years. Although the induction of tumor cell plasticity [2] in response to anti-cancer therapies has been previously detected [15], a strategy to exploit this plasticity and provide successful treatment is still lacking.

In this study we provided a novel, single-cell framework for the improved resolution of intra-tumor cellular heterogeneity, allowing for the identification of independently evolving subpopulations. High-throughput FACS data was analyzed using single-cell information-theoretic surprisal analysis. The analysis resolved unbalanced protein subnetworks in the tumor [17], which were further attributed to single cells. Each cell was assigned a cell-specific signaling signature (CSSS), composed of a set of altered subnetworks. Cells sharing the same CSSS were considered a subpopulation. This strategy not only resolved overexpressed biomarkers or altered protein-protein correlation networks in response to RT treatment, but also mapped single-cell signaling signatures within the tumor tissue. This information enabled the resolution of distinct cellular subpopulations, information that is critical for accurate treatment design. Our analysis requires only one tissue/sample to elucidate the perturbed networks operating in each tumor, where the large number of single cells analyzed (>100,000/experiment) provides a high resolution of tumor heterogeneity. This is in contrast to bulk analysis which requires large datasets comparing multiple tissues in order to reveal high-resolution altered networks in each patient [19, 20, 30]. Furthermore, CSSS-based analysis efficiently identifies small cellular subpopulations, which are likely to be missed in bulk analyses.

Using the CSSS strategy, we revealed that two distinct cellular subpopulations harboring altered subnetworks with induced Her2 and cMet proteins, respectively, expanded in TNBC tumors in response to RT. Using in vitro and in vivo murine models, human cell lines, and patient-derived TNBC, we showed that efficient sensitization of aggressive TNBC to RT could be achieved only when Her2 and cMet proteins were inhibited simultaneously. Despite the fact that the in-vivo follow-up was only up to three weeks, the results demonstrated a significant synergistic effect in tumor response to RT and combined targeted therapy, compared to RT alone. While RT-treated tumors developed resistance, the tumors pre-treated with Her2 and cMet inhibitors exhibited durable remission. In an extended follow-up period, there is a chance that other minor sub-populations may arise, which were not seen during the three weeks. In a clinical setting, a longer patient-specific follow-up might provide additional data for a more accurate treatment plan.

## Conclusions

In summary, we reveal a novel approach to resolve in-depth intra-tumor heterogeneity at the single-cell level. This strategy provides an essential step towards the accurate design of targeted drug combinations for evolving tumor resistance. We validate this strategy by elucidation and detailed analysis of TNBC plasticity that allows for the sensitization of tumors to RT.

Importantly, this approach allows for the mapping of distinct cellular subpopulations in a single tumor, without the need to be compared to and analyzed relative to other tumors, such as in the case of bulk analyses. This strategy can be universally applied to any cancer type and any treatment strategy by tailoring the panel of oncomarkers for a particular cancer type, where the computational approach would remain essentially the same. The value of this strategy will increase alongside the continued development of single-cell and mass cytometry techniques, which will allow for the simultaneous detection of dozens [58–60], possibly even hundreds, of signaling proteins in statistically significant numbers (>50,000–1,000,000) of single cells obtained from a single tumor.

## Supplementary Information

**Additional file 1.** Provides the details for the TNBC models and single cell analyses used in the study.

**Additional file 2: Supplementary information file.** Includes all the supplementary figures (Fig. S1-Fig. S7; and Table S8) with supplementary methods.

**Additional file 3.** Data file of in vitro experiments in the 4T1 system. The data includes single cell protein expression levels as measured by FACS, lambda (*λ*_*α*_(*cell*)) values, G (*G*_*iα*_) values and % of subpopulations out of the entire population.

**Additional file 4.** Data file of in vivo experiments in the 4T1 system 6 days post RT. The data includes single cell protein expression levels as measured by FACS, lambda (*λ*_*α*_(*cell*)) values and G (*G*_*iα*_) values and % of subpopulations out of the entire population.

**Additional file 5.** Data file of in vivo experiments in the 4T1 system 12 days post RT. The data includes single cell protein expression levels as measured by FACS, lambda (*λ*_*α*_(*cell*)) values and G (*G*_*iα*_) values and % of subpopulations out of the entire population.

**Additional file 6.** Data file of in vitro experiments in BR45 cells 6 and 14 days post RT. The data includes single cell protein expression levels as measured by FACS, lambda (*λ*_*α*_(*cell*)) values and G (*G*_*iα*_) values and % of subpopulations out of the entire population.

**Additional file 7.** Data file of in vivo experiments in BR45 PDX mice 6 days post RT. The data includes single cell protein expression levels as measured by FACS, lambda (*λ*_*α*_(*cell*)) values and G (*G*_*iα*_) values and % of subpopulations out of the entire population.

**Additional file 8.** Data file of in vivo experiments in BR45 PDX mice 12 days post RT. The data includes single cell protein expression levels as measured by FACS, lambda (*λ*_*α*_(*cell*)) values and G (*G*_*iα*_) values and % of subpopulations out of the entire population.

## Data Availability

All data generated or analyzed during this study are included in this published article and its supplementary information files. The codes for single-cell computational analysis are publicly available from Github [31] (https://github.com/cohenoa/Computational-quantification-of-cellular-subpopulations-within-tumors-in-anti-cancer-therapy).

## Abbreviations

RT: Radiation therapy
CSSS: Cell-specific signaling signature
SA: Surprisal analysis
Her2: Human epidermal growth factor receptor 2
cMet: Cell surface mesenchymal-epithelial transition factor
TNBC: Triple-negative breast cancer
MDA-MB-231: M.D. Anderson-Metastasis Breast cancer-231
MDA-MB-468: M.D. Anderson-Metastasis Breast cancer-468
FACS: Fluorescence-activated single-cell sorting
EGFR: Epidermal growth factor receptor
EpCAM: Epithelial cell adhesion molecule
CD44: Cell surface glycoprotein
CD24: Small cell lung carcinoma cluster 4 antigen
PD-L1: Programmed cell death 1 ligand 1
KIT: Proto-oncogene, receptor tyrosine kinase
CD133: Prominin 1-CD133 antigen
E-Cadherin: Calcium-dependent adhesion protein, epithelial
MUC1: Tumor-associated epithelial mucin
MB: Methylene blue
MTT: 3-(4,5-dimethylthiazol-2-yl)-2,5-diphenyl-2H-tetrazolium bromide, used to assess cell viability as a function of redox potential
