# Supplementary material for: Computational quantification and characterization of independently evolving cellular subpopulations within tumors is critical to inhibit anti-cancer therapy resistance
Source: Genome Med. 2022 Oct 20;14:120. doi: 10.1186/s13073-022-01121-y (PMC9583500; doi:10.1186/s13073-022-01121-y)
Supplement: Supplementary file 2 — Additional file 2: Supplementary information file. Includes all the supplementary figures (Fig. S1-Fig. S7; and Table S8) with supplementary methods. [file 13073_2022_1121_MOESM2_ESM.docx]

**Supplementary file**

**Computational quantification and characterization of independently evolving cellular subpopulations within tumors is critical to inhibit anti-cancer therapy resistance**

Heba Alkhatib^1#^, Ariel M. Rubinstein^1#^, Swetha Vasudevan^1^, Efrat Flashner-Abramson^1^, Shira Stefansky^1^, Sangita Roy Chowdhury^1^, Solomon Oguche^1^, Tamar Peretz-Yablonsky^2^, Avital Granit^2^, Zvi Granot^3^, Ittai Ben-Porath^3^, Kim Sheva^4^, Jon Feldman^2^, Noa E. Cohen^5^, Amichay Meirovitz^4^**^*^** and Nataly Kravchenko-Balasha^1^**^*^**

^#^ Equal contribution

**^*^** Equal contributors and corresponding authors

^1^ The institute of Biomedical and Oral Research, The Hebrew University of Jerusalem, Jerusalem, Israel.

^2^ Sharett Institute of Oncology, Hebrew University-Hadassah Medical Center, Jerusalem, Israel.

^3^ Department of Developmental Biology and Cancer Research, Institute for Medical Research-Israel-Canada, The Hebrew University-Hadassah Medical School, 91120, Jerusalem, Israel.

^4^ The Legacy Heritage Oncology Center & Dr. Larry Norton Institute, Soroka University Medical Center, Ben Gurion University of the Negev, Faculty of Medicine, 8410101, Beer Sheva, Israel.

^5^ School of Software Engineering and Computer Science, Azrieli College of Engineering, Jerusalem, 9103501, Israel.

To whom correspondence may be addressed. Email: N.K.B: natalyk@ekmd.huji.ac.il or A.M: amichaym@gmail.com

[Supplementary Methods 3](#_Toc516046972)

[Cell maintenance 3](#_Toc516046976)

[Irradiation of TNBC cells. 3](#_Toc516046977)

[Flow Cytometry. 4](#_Toc516046979)

[Mice models and tumor inoculation. 5](#_Toc516046978)

[In-vivo treatments. 6](#_Toc516046979)

[Western Blot. 6](#_Toc516046979)

[Supplementary Figures and legends 7](#_Toc516046980)

[Fig. S1. 7](#_Toc516046981)

[Fig. S2. 9](#_Toc516046982)

[Fig. S3. 11](#_Toc516046983)

[Fig. S4. 12](#_Toc516046984)

[Fig. S5. 13](#_Toc516046985)

[Fig. S6. 15](#_Toc516046984)

[Fig. S7. 16](#_Toc516046985)

[Supplementary Tabel 17](#_Toc516046980)

Table S8 17

[References 18](#_Toc516046980)

**Supplementary Methods**

**Cell maintenance.**

4T1 cells were maintained in Dulbecco's modified Eagle's medium (DMEM), MDA-MB-231 and MDA-MB-468 cells were maintained in RPMI-1640 medium, both supplemented with 10% FBS, 4 mM L-glutamine, 100 U/mL Penicillin and 100 μg/mL Streptomycin. All media and supplements were purchased from Biological Industries, Israel. All cell lines were maintained at 37 °C in 5% CO_2_. Cells were checked on a routine basis to ensure the absence of mycoplasma contamination.

**Irradiation of TNBC cells.**

4T1, MDA-MB-231, MDA-MB-468 and BR45 cells were trypsinized and plated to reach optimal confluence by the next day (70-80%) before irradiation treatment. 4T1 cells were irradiated with 5 and 15 Gy of γ-rays using a radiotherapy unit (gamma cell 220) at a dose rate of 1.5 Gy/min. Radiation doses were selected based on calibration experiments in which the cellular survival rates after irradiation ranged from 40-50%. MDA-MB-231 and BR45 cells were treated with 10 Gy, and MDA-MB-468 cells were treated with 5 Gy. Afterwards, cells were grown under normal conditions for 24h, 48h and 6 days. At each indicated time point, cells were detached from the flask using *Accutase* and fixed using 2% paraformaldehyde for 30 min on ice. The labelling procedure for each condition performed on the day of the flow cytometry analysis was as mentioned below.

**Preparation of single cell suspensions using mechanical methods.** The harvested tumors were washed twice with PBS at RT and mechanically dissosiated thoroughly using magnetic stirrer apparatus. The masses were then gently mashed with the back of a 10 ml plastic syringe for mechanical digestion. After being placed in the stir apparatus for 15-20 min, the tumor/PBS buffer mixture was strained using a 70µm cell strainer, centrifuged for 5 min at 3000 rcf and resuspended in FACS buffer.

**Red blood cells lysis.** To lyse RBCs from freshly harvested tumors, cells were re-suspended in 10 ml RBC lysis buffer (0.8 g NH4CL + 0.1 g KHCO3 in 100 ml DDW) for 5 min. at RT. To stop the reaction of the buffer, 30 ml of DMEM supplemented with 10% FBS was added and the mixture was centrifuged for 5 min at 3000 rcf to remove the lysis buffer.

**Fixation of cells.** Samples were fixed with 2% PFA (#15710, EMS ) for 30 min on ice.

## **Flow Cytometry.**

The following fluorescently tagged antibodies, were obtained from BioLegend, Inc.: EpCAM (9C4/G8.8), CD45 (2D1/104), CD31(WM95/390), CD140a (16A1/APA5), CD44 (IM7), E-Cadherin (DECMA-1), EGFR (AY13), CD24 (M1/69), CD24 (ML5), KIT (ACK2/104D2), CD133 (315-2C11/clone7), PD-L1 (10F.9G2/29E.2A3). ERBB2 / Her2 (5J297) was obtained from LifeSpan BioScience. Anti-MUC1 Polyclonal Antibody and Anti-Met Polyclonal Antibody were both obtained from Bioss Antibodies Inc. (See Table S8).

**Blocking of Endogenous** **Fc.** 1.5 ml centrifuge tube, each containing 0.8×10^6^ cells were incubated for 30 mins on ice with 50 µl of Fc blocker buffer (FACS Buffer + Fc Blocker: anti-mouse CD16/32 Antibody, Biolegend #101301, 1:50).

**Labelling Procedure.** Each sample was labelled with 11 fluorescently tagged Ab mixture. (Table S8). A cocktail of 3 additional Abs with the same fluorophore (PE) was used to exclude hematopoietic (CD45), fibroblast (CD140) and endothelial cells (CD31), to ensure that only tumor cells were analyzed later on. This exclusion is not required in the case of staining parental cells which do not consist of the tumor microenvironment. The 11 fluorescently labelled Abs are detailed in Table S8. An unstained control sample for each condition was used along with a single color control for each Ab using UltraComp Compensation eBeads**™** according to the manufacturer's instructions for creating compensation controls. The labelling time was extended to 40 min on ice in the dark. Samples were washed with FACS buffer, centrifuged and resuspended in 700 μl of flow FACS buffer, then filtered right before reading with LSR-Fortessa Analyzer into FACS tubes. ~50,000 cells were profiled for each sample.

**Preliminary data analysis***.* The preliminary data analysis was done using FlowJo VX software. The output data were extracted into an excel file in which each row represented a single cell and each column showed the intensity of each assayed protein (FCS Extract 1.02 software). The level of fluorescence of 97% of unstained cells (mainly due to autofluorescence) were used to define the minimal limits of true fluorescent staining of the cells. All the values equal or below these limits were converted to arbitrary low positive values of [=10] for further analysis.

**Mice models and tumor inoculation.**

4T1 mouse breast carcinoma mimics stage IV triple negative breast cancer in humans. Tumors were harvested after euthanizing female mice. The time elapsed from tumor inoculation varied from 2 weeks to 1 month. Patient-derived TNBC BR45 cells were used to establish PDX models, tumors were harvested 2 months after orthotopic implanting of xenografts in the NSG mouse model.

**In-vivo treatments.**

Targeted inhibitors: Trastuzumab was given IP twice a week with a concentration of 5mg/kg, and the vehicle used was 200μl sterile saline. Crizotinib and erlotinib were given by gavage with a concentration of 25 mg/kg and 12.5 mg/kg respectively, five consecutive days a week. The vehicle used was hydroxypropyl methylcellulose with 0.2% tween. Mice were treated for 3 weeks. During this period the tumors volumes were measured regularly to observe the effect of the drug.

**Western blot analysis.**

Antibodies: Western blot antibodies were obtained from Cell Signaling Technology, Inc.: anti-phospho-Akt (Thr308, #4056S), anti-phospho-Akt (Ser473, #9271S), anti-total-Akt (#4691S), anti-phospho-ERK1/2 Thr202/Tyr204 (#9101S), anti-total-ERK1/2 (#9102S), anti-cleaved PARP(#5625S), Cleaved Caspase-3 (Asp 175, #9661S), Phospho-S6 Ribosomal Protein (ser235/236, #2211S) (D57.2.2E) XP® Rabbit mAb. GAPDH Antibody (#32233) was obtained from Santa Cruz Biotechnology Inc.

**Supplementary Figures and legends**

**Fig. S1**

**
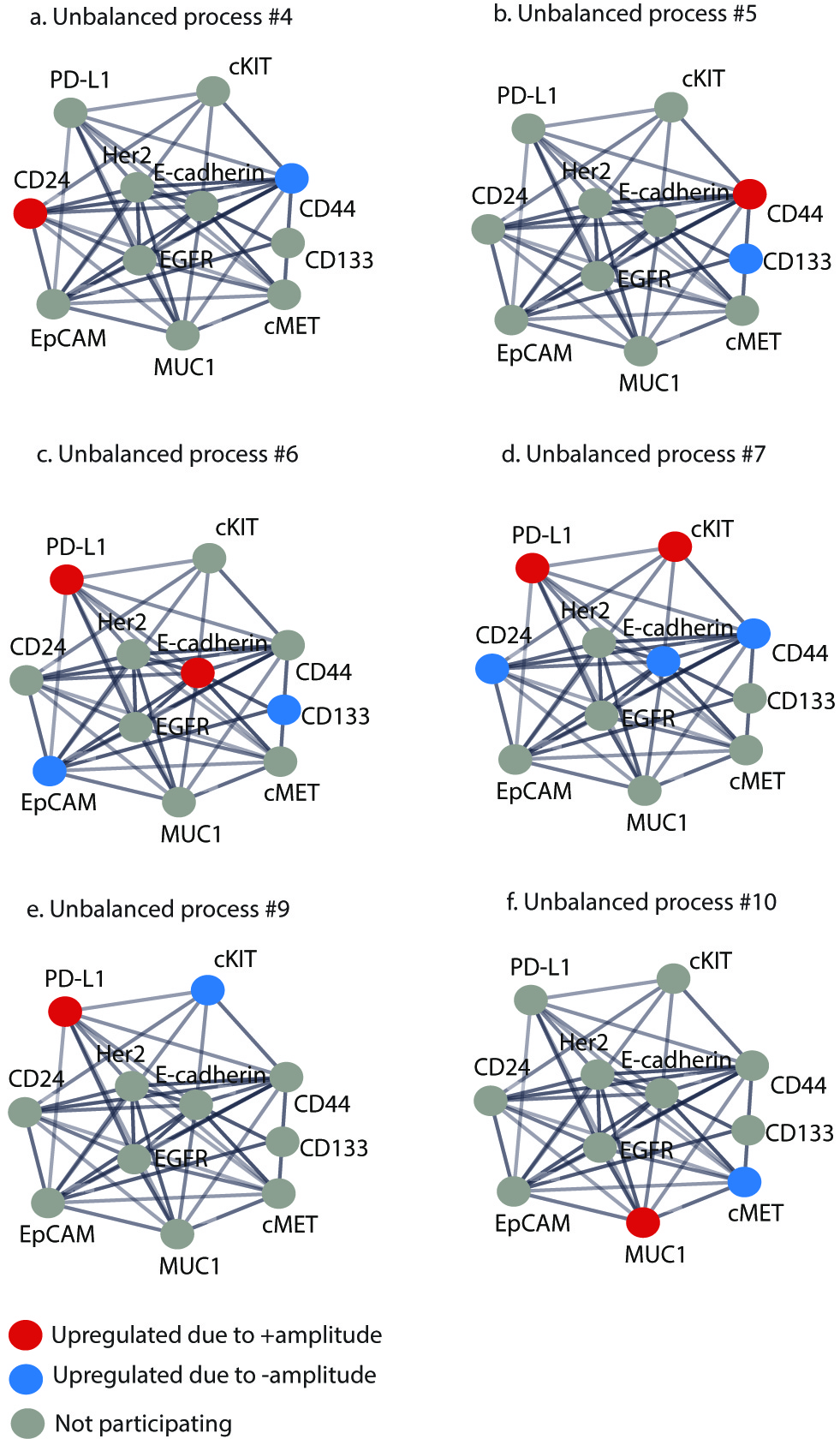
**

**Fig. S1 Unbalanced processes (=subnetworks) $\alpha=4-10$ as identified by surprisal analysis for 4T1 irradiated cells.** For every process *α*, the proteins with significant *  * values were assembled into subnetworks. The colors in this figure indicate correlation or anticorrelation (blue is anticorrelated with red). Upregulation or downregulation due to the process can be defined further using a product for each protein in every *cell* in each experimental condition/time point (as it is done in Figure 3). All proteins involved in the process were assigned connections using STRING database^1^.

##

**
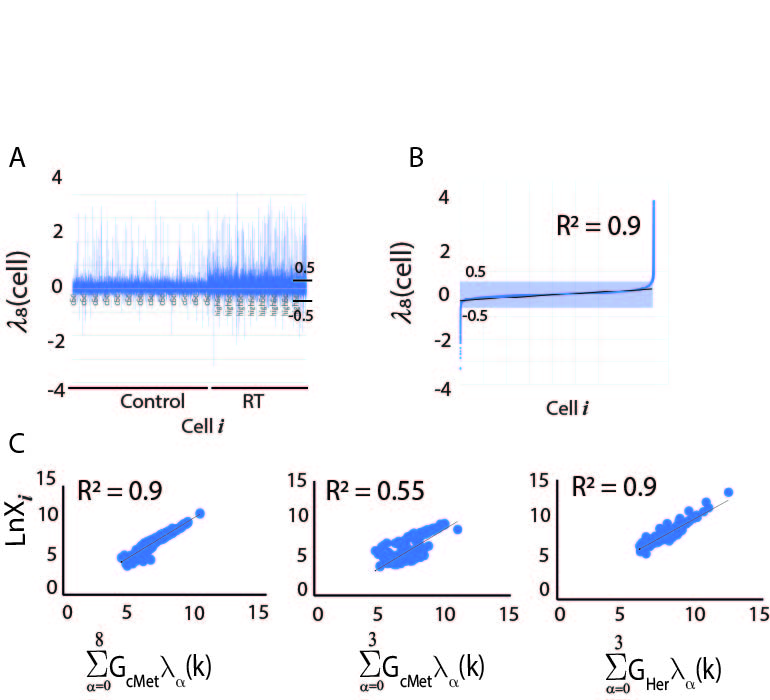
Fig. S2**

**Fig. S2 Data reproducibility and limit values. (A)** A plot representation of values for process 8 in 4T1 cells is shown as an example. The amplitudes of unbalanced process 8 are plotted for each untreated and irradiated cell (15Gy) 6d post treatment. (**B**) Sorted values of the 4T1 single cell data, including all conditions, are presented. The lambda values are considered significant when they are located on the definitive tails. In other words, the values located within the blue box (< 0.5 or > -0.5) change continuously, generating a linear curve with a meaningful R^2^ >0.9. However values falling outside of the blue box >0.5 or < -0.5) significantly change the value of R^2^ to 0.68. These values, which deviate significantly from those values close to 0, are considered for further analysis. The same thresholds are kept in all conditions. **(C)** To verify that a protein *i* is influenced by a particular process/processes, we plot against $\ln X_{i}(k)$for different proteins, and for different values of n, and examine the correlation between them as n is increased. An unbalanced process, $\alpha=n$, was considered significant if it significantly improved the correlation. Figure S2 exemplifies this process for Her2 and cMet, showing that increasing n to n=8 (which includes process 8) affects the correlation and strongly reproduces the experimental data of cMet for cells with * *values >0.5 (Fig S2. C, left panel). However, the reproducibility of cMet expression levels decreased significantly for the same cells when only the first three processes, including Her2+ process 3, are considered (Fig S2. C, middle panel). This indicates that in these cells, the first three processes are not enough to achieve high correlation ( <0.9) between the theoretical and experimental data. In the case of Her2, a high correlation was achieved when only the first three processes were considered (Fig S2. C, right panel) in the cells harboring process 3.

**
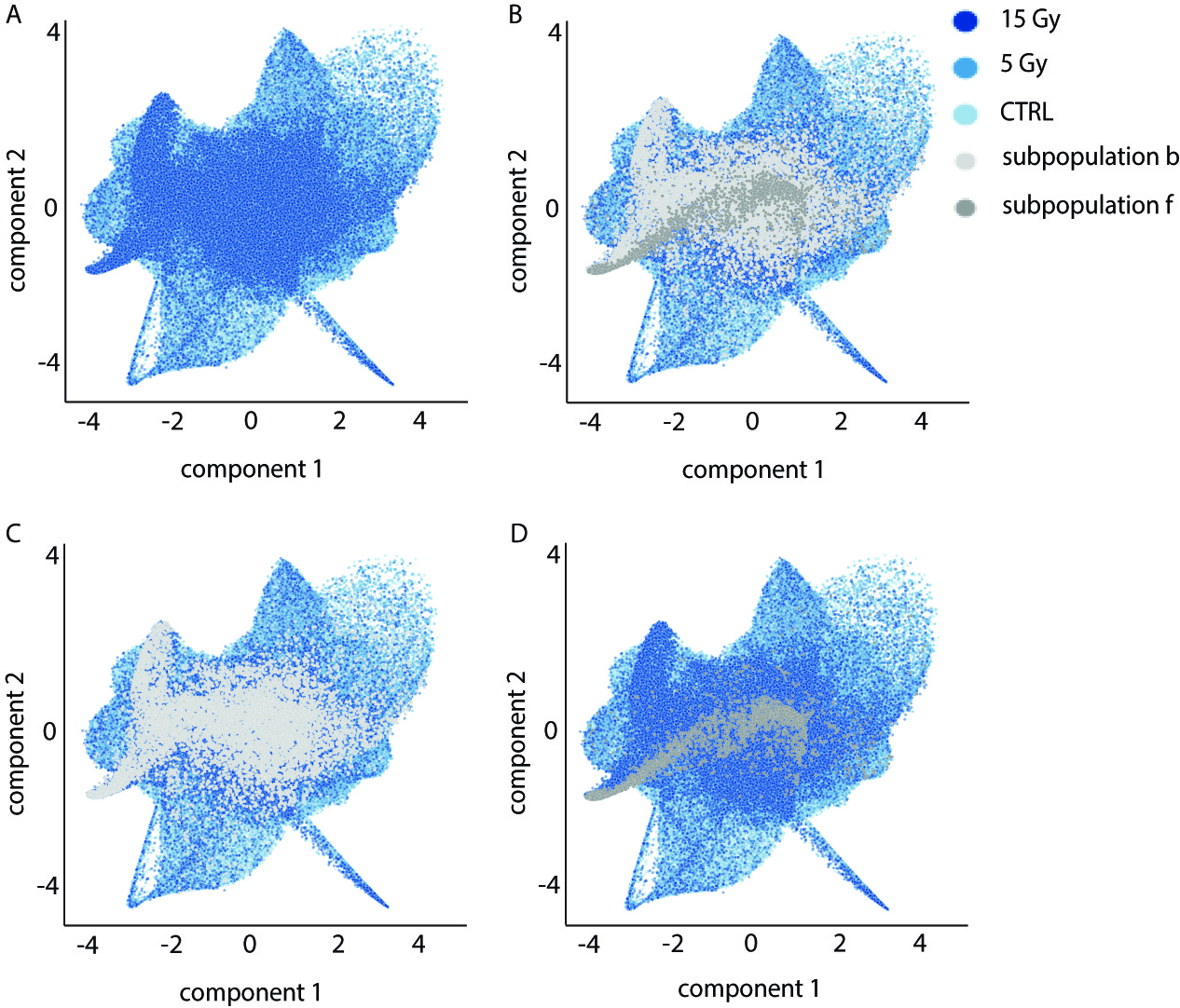
 Fig. S3**

**Fig. S3 t-SNE analysis of the 4T1 single cell data. (A)** t-SNE analysis of 4T1 cells irradiated with 5Gy or 15Gy. **(B)** Subpopulations b and f, harboring processes 3 and 8 respectively, (as identified using CSSS analysis) were mapped onto the t-SNE plot showing the two separate subpopulations that expanded in response to RT. **(C)** and (**D)** respectively show subpopulations**b**and**f** on separate t-SNE plots. The tSNE parameters used were: learning rate = 200 and perplexity = 1000.

**
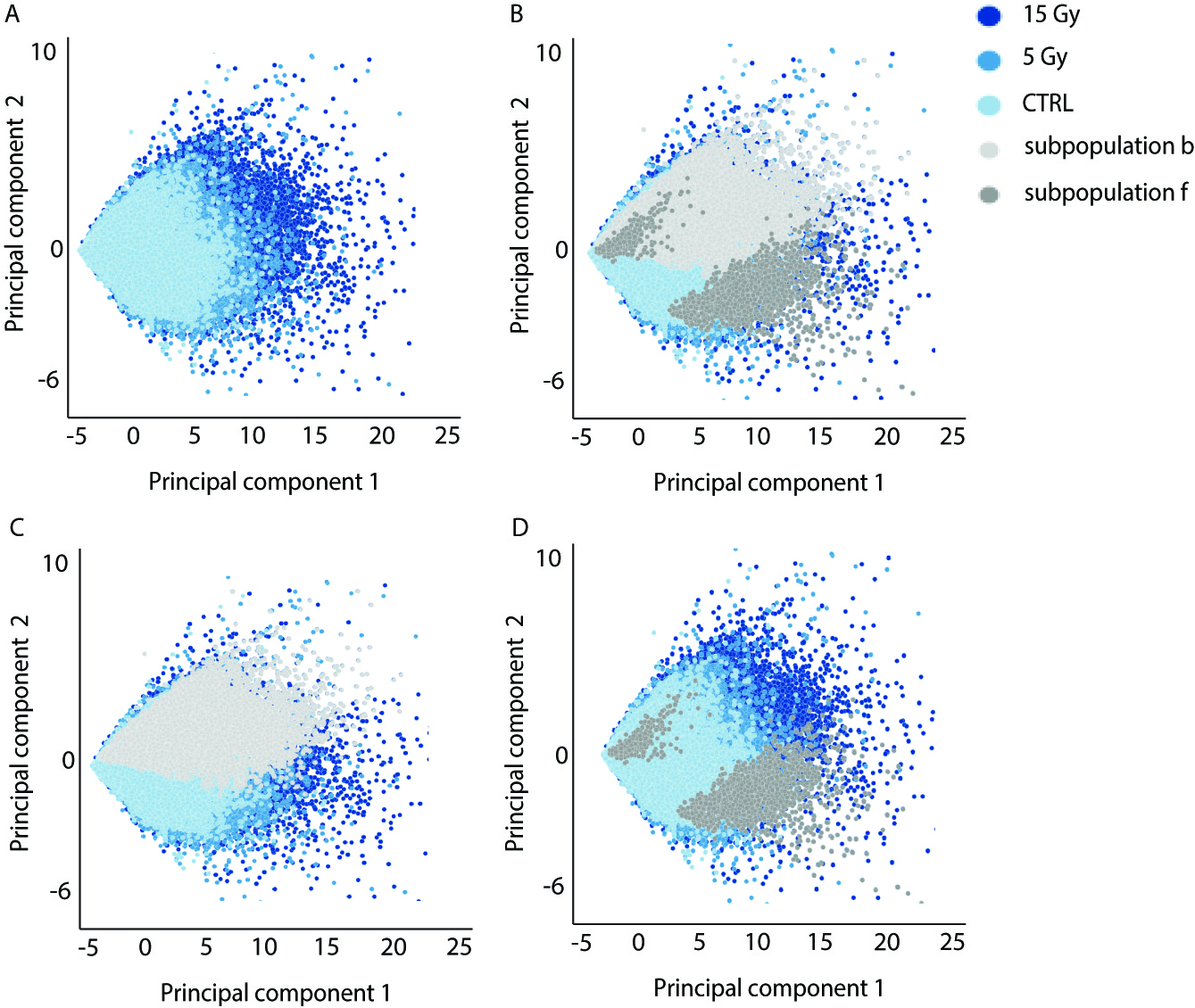
Fig. S4**

**Fig. S4 PCA analysis of 4T1 single cell data. (A)** 4T1 cells were irradiated with 5Gy or 15Gy. Principal component analysis of the data shows minority separation between the control and RT treated cells in the PC1/PC2 plot. (**B**) Subpopulations **b** and **f**, harboring processes 3 and 8 respectively, (as identified using CSSS analysis) were mapped onto the PCA plot, which shows how the generation of two separate subpopulations expanding in response to RT cannot be discerned using PCA. **(C)** and (**D)** respectively show subpopulations**b**and**f** on separate PCA plots.

**
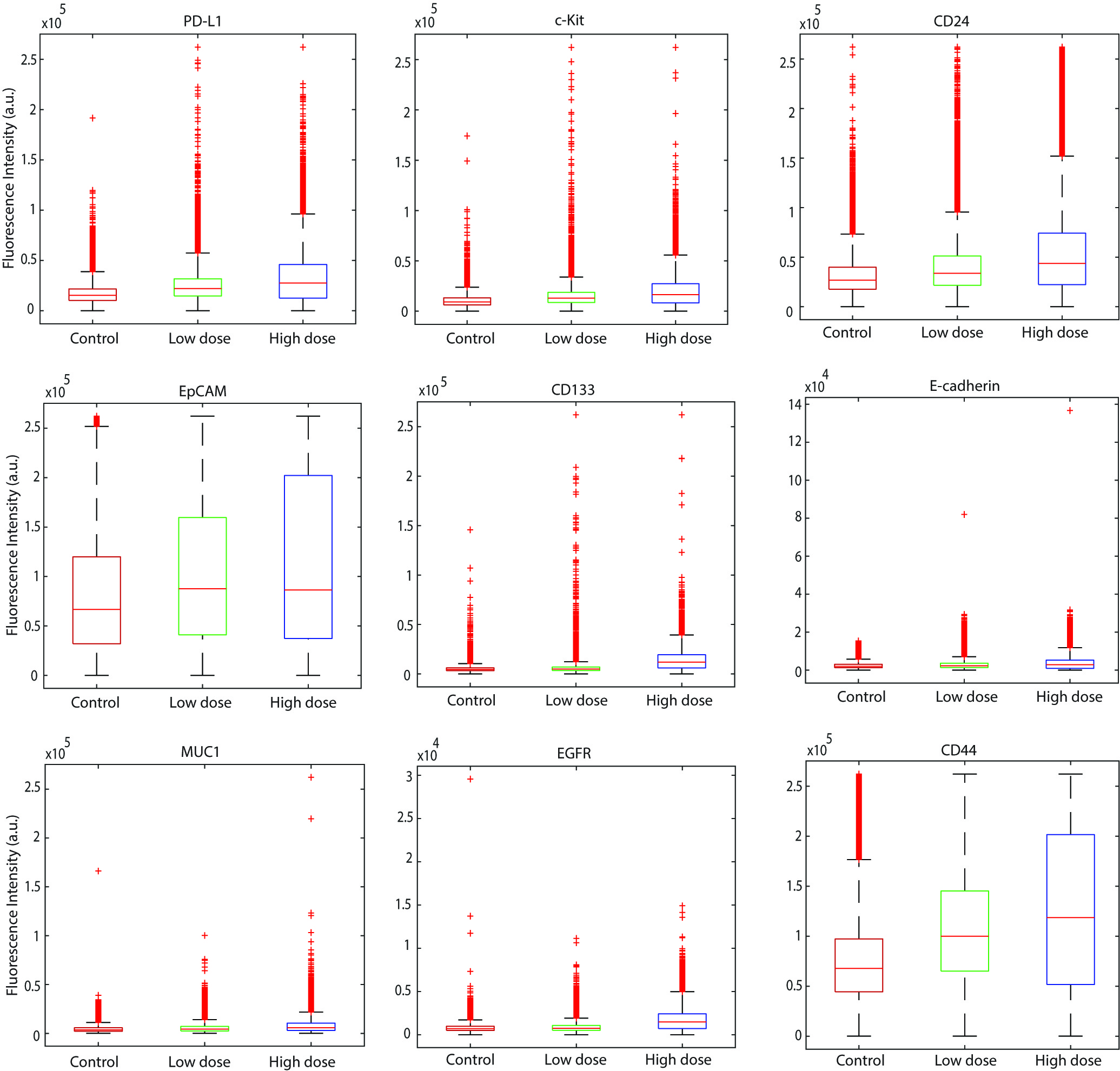
Fig. S5.**

**Fig. S5 Expression levels of 9 proteins (EGFR, EpCAM, CD44, CD24, PD-L1, KIT, CD133, E-Cadherin, and MUC1) before and after irradiation in 4T1 cells.** Raw FACS data of protein expression levels in response to low dose (5 Gy) or high dose (15 Gy) are shown as one-dimensional boxplots.

**
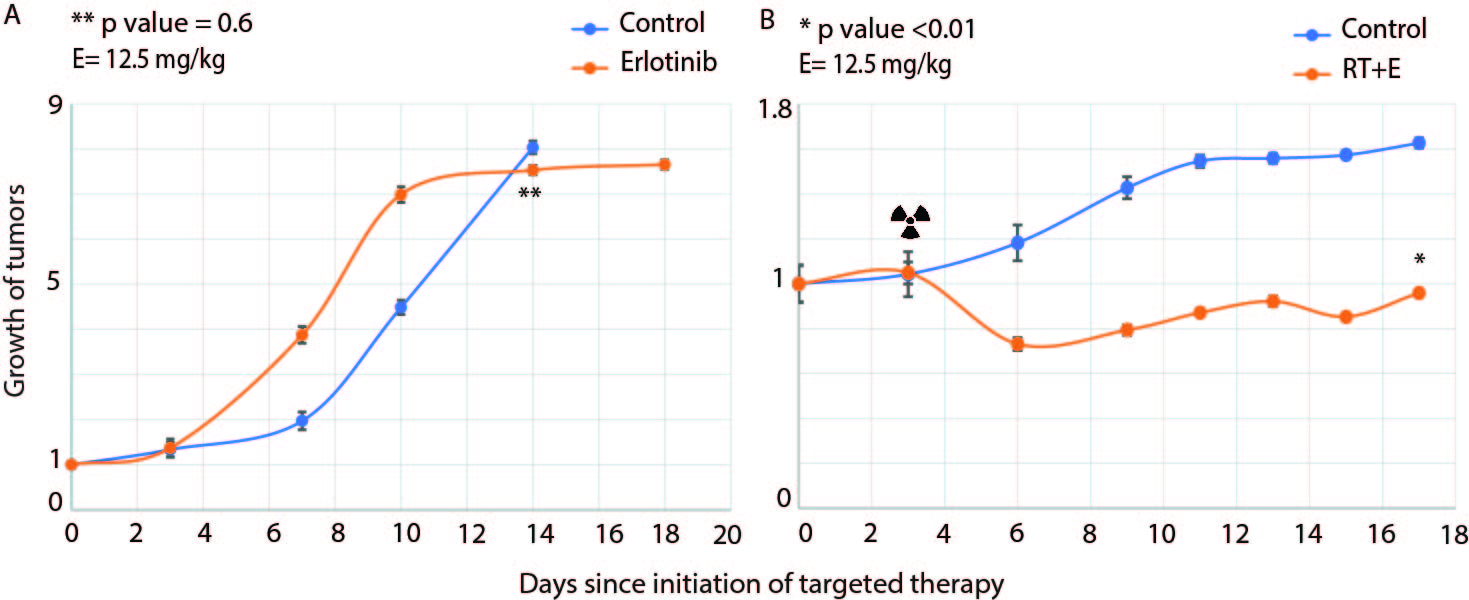
Fig. S6**

## **Fig. S6** **EGFR inhibition sensitizes BR45 PDX to RT similarly to anti-Her2 monotherapy**. Complementary information for Figure 6. Effect of Erlotinib alone (**A**) and RT+E (RT + erlotinib, **B**) is demonstrated using BR45 tissues. TNBC BR45 tumor tissues were transplanted orthotopically into NSG mice, treated with brachytherapy on days 3 and 5 with 12 Gy and 10 Gy respectively. Erlotinib was administrated from d0 (3 days prior to RT) until the end of the experiment (d17); ± S.E. and p values are shown. Normalized tumor volumes are shown (tumor volumes were divided by control volume values for the 1st time point).

#
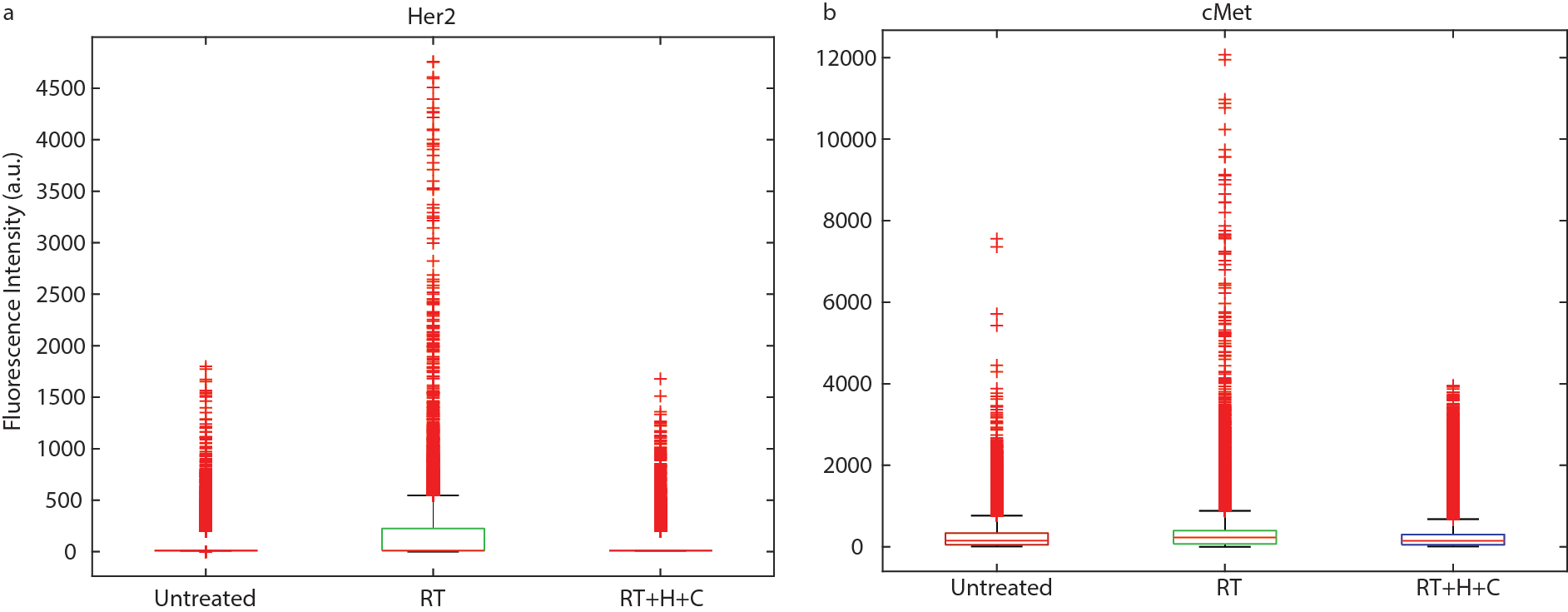
 **Fig. S7**

**Fig. S7 In-vivo change in the expression levels of Her2 and cMet in BR45 cells.** One-dimensional boxplots show the upregulation of Her2 and cMet after irradiation on two alternative days with (12, 10) Gy respectively and the downregulation of these proteins when the CSSS-predicted targeted therapy was applied.

# **Table S8. Antibodies for flow cytometry analysis.**

| Protein | Reactivity | Color | Company | Cat. # | Concentration μl/(8×10**^5^** cells) |
| --- | --- | --- | --- | --- | --- |
| CD45 | Mouse (M) | PE | Biolegend | 103105 | 0.2 |
| CD140 | M | PE | Biolegend | 135905 | 1 |
| CD31 | M | PE | Biolegend | 102407 | 0.3 |
| CD326 – EpCAM | M | APC | Biolegend | 118213 | 1 |
| CD24 | M | BV421 | Biolegend | 101825 | 0.75 |
| CD117 – cKit | M | BV650 | Biolegend | 135125 | 1.5 |
| CD274 – PD-L1 | M | BV605 | Biolegend | 124321 | 1.5 |
| CD133 | M | PE/Dazzel594 | Biolegend | 141211 | 1.5 |
| CD45 | Human (H) | PE | Biolegend | 638512 | 0.2 |
| CD140 | H | PE | Biolegend | 323504 | 1 |
| CD31 | H | PE | Biolegend | 303405 | 0.25 |
| CD326 – EpCAM | H | APC | Biolegend | 324207 | 1 |
| CD24 | H | BV421 | Biolegend | 311121 | 0.75 |
| CD117 – cKit | H | BV650 | Biolegend | 313221 | 1.5 |
| CD274 – PD-L1 | H | BV605 | Biolegend | 329723 | 1.5 |
| CD133 | H | PE/Dazzel594 | Biolegend | 372811 | 1.5 |
| CD44 | H/M | BV510 | Biolegend | 103043 | 1.5 |
| cMet | H/M | Alexa Fluor 750 | Bioss | 0668R-A750 | 1.5 |
| MUC1 | H/M | Alexa Fluor 680 | Bioss | 1497R-A680 | 1.5 |
| Her2 | H/M | FITC | LifeSpan BioScience | C533753 | 1 |
| EGFR | H/M | Per/CP5.5 | Santa Cruz | 20 PCPC5 | 1 |
| CD324 – Ecad | H/M | PE/Cy7 | Biolegend | 147309 | 1.5 |

# **References**

1 Szklarczyk D *et al.* The STRING database in 2011: functional interaction networks of proteins, globally integrated and scored. *Nucleic Acids Res* 2011; **39**: D561-8.
